# The abortion and mental health controversy: A comprehensive literature review of common ground agreements, disagreements, actionable recommendations, and research opportunities

**DOI:** 10.1177/2050312118807624

**Published:** 2018-10-29

**Authors:** David C Reardon

**Affiliations:** Elliot Institute, Springfield, IL, USA

**Keywords:** Abortion mental health, abortion, reproductive health, post-abortion trauma, research bias, complicated grief, ambiguous loss, bereavement, post-traumatic stress disorder, pregnancy loss

## Abstract

The abortion and mental health controversy is driven by two different perspectives regarding how best to interpret accepted facts. When interpreting the data, abortion and mental health proponents are inclined to emphasize risks associated with abortion, whereas abortion and mental health minimalists emphasize pre-existing risk factors as the primary explanation for the correlations with more negative outcomes. Still, both sides agree that (a) abortion is consistently associated with elevated rates of mental illness compared to women without a history of abortion; (b) the abortion experience directly contributes to mental health problems for at least some women; (c) there are risk factors, such as pre-existing mental illness, that identify women at greatest risk of mental health problems after an abortion; and (d) it is impossible to conduct research in this field in a manner that can definitively identify the extent to which any mental illnesses following abortion can be reliably attributed to abortion in and of itself. The areas of disagreement, which are more nuanced, are addressed at length. Obstacles in the way of research and further consensus include (a) multiple pathways for abortion and mental health risks, (b) concurrent positive and negative reactions, (c) indeterminate time frames and degrees of reactions, (d) poorly defined terms, (e) multiple factors of causation, and (f) inherent preconceptions based on ideology and disproportionate exposure to different types of women. Recommendations for collaboration include (a) mixed research teams, (b) co-design of national longitudinal prospective studies accessible to any researcher, (c) better adherence to data sharing and re-analysis standards, and (d) attention to a broader list of research questions.

## Introduction

In 1992, the *Journal of Social Issues* dedicated an entire issue to the psychological effects of induced abortion. In an overview of the contributors’ papers, the editor, Dr Gregory Wilmoth, concluded,

There is now virtually no disagreement among researchers that some women experience negative psychological reactions postabortion. Instead the disagreement concerns the following: (1) The prevalence of women who have these experiences …, (2) The severity of these negative reactions …, (3) The definition of what severity of negative reactions constitutes a public health or mental health problem …, [and] (4) The classification of severe reactions …^1^

Twenty-six years later, the body of literature has grown. Today, there are many additional areas of agreement, but the areas of disagreement have also grown.

As with most controversies, the abortion and mental health (AMH) controversy is driven by at least two different perspectives regarding how best to interpret accepted facts. A useful parallel is found in the debate over climate change. On the fringes of the climate change controversy are non-experts who hold an extreme position of either total denial or total credulity. But it is far more common for skeptics to acknowledge that fossil fuels make some contribution to global warming while still arguing that these effects are not as extreme global warming proponents contend.^2^ This group may be described as global warming minimalists. Their normal pattern is to interpret the data in a way that minimizes the potential threat. By contrast, global warming proponents may be more likely to interpret the data in ways that emphasize the potential risks.

Similarly, in regard to the AMH controversy, there are both AMH minimalists and AMH proponents. The experts from both groups can report similar findings from the same data but will do so in ways that seem to either minimize or emphasize the negative outcomes associated with abortion. It should be carefully noted that there is actually a broad spectrum of expert views regarding the AMH link.^3^ While each researcher and expert has likely developed carefully considered and nuanced opinions, these have not been completely disclosed and cannot be cataloged in regard to every issue discussed herein. Still, broadly speaking, it is evident that both expert reviews and the authors of individual studies appear to generally support either the view that (a) the mental health effects associated with abortion are minimal and within the expected range for the women seeking abortions^4–10^ or (b) the effects are significant enough to justify more research dollars, and better screening and counseling in order to reduce the number of adverse outcomes.^11–19^ In addressing this conflict, it is not my intention to pigeonhole any particular expert’s viewpoint at any location on the spectrum of views regarding AMH.

In writing this review, I have tried to be as objective and fair as possible. Yet, as discussed later, since my own informed opinion is also influenced by my own experiences and preconceptions, full disclosure requires that I acknowledge at the outset that I fit most closely under the category of an AMH proponent. That said, my goal is not to dismiss or disprove the viewpoint of “the other side,” but rather to understand and engage with it in a manner that will contribute to a respectful “transformational dialogue” that will help to “crystalize the areas of agreement and disagreement along with opportunities for collaboration.”^20^ In this regard, it is my great hope that those who disagree with my analysis and conclusions herein will use the publication of this review as an opportunity to publish responses and reviews that address the issues raised with additional depth from their perspectives.

The method I used for this review was to carefully examine previous literature reviews regarding mental health effects associated with legal abortion that have been published since 2005.^4–10,12–19,21,22^ In that sense, this article may be considered a review of reviews of the literature on AMH. In addition, I studied the references cited in these various reviews in order to further my effort to more completely identify (a) areas of agreement and disagreement, (b) the underlying reasons for disagreements, and (c) opportunities to collaborate in light of the current literature.

This undertaking is intended to advance more than just an academic discussion, however. Research has shown that women considering abortion have a high degree of desire for information on “all possible complications,” including rare risks.^23^ Therefore, an updated and more complete understanding of the literature can and should better prepare physicians and mental healthcare providers with more accurate and helpful information for advising and counseling women before or after an abortion. For example, better screening for risk factors should help to identify women who may benefit from additional pre- or post-abortion counseling^24–38^ and may also help to prevent cases of women being pressured into unwanted abortions. In addition, more complete insights may help mental health counselors to be more aware and sensitive to providing the counseling services that women want and need.

This review is organized into three sections. The first examines major areas of agreement and offers a synthesis of the findings from major studies. The second section investigates the obstacles to building a consensus between AMH minimalists and AMH proponents, including institutional and ideological biases, research obstacles, poorly defined terms, and similar issues that contribute to the disparity in the conclusions most emphasized by each side. The third section provides recommendations for collaborative research based on the insights gained from the first two sections, addressing such issues as data sharing, mixed research teams, and how to maximize the value of longitudinal prospective studies.

## Areas of agreement

### Abortion contributes to negative outcomes for at least some women

The 2008 report of the American Psychological Association’s (APA) Task Force on Mental Health and Abortion (TFMHA) concluded that “it is clear that some women do experience sadness, grief, and feelings of loss following termination of a pregnancy, and some experience clinically significant disorders, including depression and anxiety.”^4^ Indeed, task force chair Brenda Major et al.’s^39^ own research had reported that 2 years after their abortions, 1.5% of the remnant participating in her case series (38% of the 1177 eligible women, after dropouts) had all the symptoms for *abortion-specific* post-traumatic stress disorder (PTSD). In addition, she found that compared to their 1-month post-abortion assessments, at 2 years the participating remnant had significantly *rising rates* of depression and negative reactions and lowering rates of positive reactions, relief, and decision satisfaction.^39^

The fact that some women do have maladjustments is most specifically documented in case studies developed by post-abortion counselors successfully treating women with maladjustments, including counselors working from a pro-choice perspective^40–44^ as well as from those working from a pro-life perspective.^45–47^

Even one of the harshest critics of the “myth” of abortion trauma, psychiatrist Nada L Stotland,^40^ subsequently reported her own clinical experience treating a patient whose miscarriage triggered a mental health crisis arising from unresolved issues regarding a prior abortion. Stotland, who later served as president of the American Psychiatric Association, subsequently began to recommend screening of prospective abortion patients for risk factors in order to guide decision counseling and identify additional counseling needs.^31^

### Some groups of women are predictably at greater risk of negative outcomes

There is a strong research-based consensus that there are numerous risk factors that can be used to identify which women are at greatest risk of negative psychological outcomes following one or more abortions. Indeed, the TFMHA concluded that one of the few areas of research which can be most effectively studied is in regard to efforts to “identify those women who might be more or less likely than others to show adverse or positive psychological outcomes following an abortion.”^4^

The TFMHA itself identified at least 15 risk factors for increased risk of negative reactions. While the TFMHA did not report on the percentage of women exhibiting each risk factor, Table 1 provides ranges of the incidence of each TFMHA risk factor as reported in the literature. The incidence rates shown in Table 1 clearly suggest that the majority of women seeking abortion have one or more of the TFMHA identified risk factors. Since exposure to multiple abortions is one of the risk factors, that risk factor alone applies to approximately half of all women having abortions, at least in the United States.^64^

**Table 1.** Risk factors for mental health problems after an abortion identified by the American Psychological Association’s Task Force on Mental Health and Abortion (TFMHA) in 2008.

TFMHA identified risk factors	Percentage of women at risk
Perceived pressure from others to terminate a pregnancy	20%;^48^ 23%;^38^ 32%;^49^ 64%^50^
Terminating a pregnancy that is wanted or meaningful	30%–63%;^48^ 26%–39%;^38^ 11%–56%;^51^ 25% fetus human, taking life;^52^ 50.7% morally wrong^50^
Perceived opposition to the abortion from partners, family, and/or friends	10%–20%^38^
Lack of perceived social support from others	44%^38^
Feelings of stigma; perceived need for secrecy	47%–56%^53^
Exposure to antiabortion picketing	87%^54^
Low perceived or anticipated social support for the abortion decision	Percent at risk not reported^55,56^
A prior history of mental health problems	31%–51%^57^
Personality factors such as low self-esteem and low perceived control over her life	53%^51^
Use of avoidance and denial coping strategies	19%–51%;^58^ 17%;^59^ 75%^60^
Feelings of commitment to the pregnancy	15%–18%;^50^ 30%^48^
Ambivalence about the abortion decision	38%–54%;^50^ 30%–44%;^61^ 65%; ^62^22%; ^63^11%–29%;^38^ 35%^48^
Low perceived ability to cope with the abortion prior to its occurrence	36%;^38^ 40%^51^
A history of prior abortion	48%–52%^64^
Abortion after the first trimester	9%^65^

Notably, the TFMHA list used here is one of the shortest that has been developed. A similar, but longer list is published in the text book on abortion most highly recommended by the National Abortion Federation.^66^ A more recent systematic search of the literature for risk factors associated with elevated rates of psychological problems after abortion cataloged 119 peer reviewed studies identifying 146 individual risk factors which the author grouped into 12 clusters.^35^ Yet another major review of risk factors identified risk factors from 63 studies which were grouped into two major categories.^25^ The first category includes 22 risk factors related to *conflicts or defects in the decision-making process*, for example, feeling pressured to abort, conflicting maternal desires and moral beliefs, and inadequate pre-abortion counseling. The second category contains 25 risk factors related to *psychological or developmental limitations*, such as pre-existing mental health issues, lack of social support, and prior pregnancy loss.^25^

The ability to identify women who are at greater risk of negative reactions has resulted in numerous recommendations for abortion providers to screen for these risk factors in order to provide additional counseling both before an abortion, including decision-making counseling, and after an abortion.^24,25,31,66–68^

Notably, while there is no dispute regarding the abundance of research identifying risk factors, there is little if any research identifying which women, if any, acquire any mental health benefits from abortion compared to carrying a pregnancy to term, even if the pregnancy was unintended or unwanted.^17^

### All AMH studies have inherent limitations

It is impossible to conduct randomized double-blind studies to investigate abortion-associated outcomes. Such studies would require random selection of women to have abortions.

Notably, the very same fact that would make such a study unethical—forcing a group of women to have abortions—actually occurs in the real world wherein some women feel pressured or even forced into unwanted abortions by their partners, parents, employers, doctors, or other significant persons.^25,45^ This problem with coerced abortions highlights one of the major difficulties involved in AMH research: any sample based entirely on self-selection (voluntary participation) no longer represents the full population of women actually having abortions. Indeed, since feeling pressured to abort is a major risk factor, the practice of excluding women aborting intended pregnancies from AMH studies^39,69^ makes the results from such studies less generalizable to the actual population of all women having abortions.

This is just one of many difficulties which makes it truly impossible to conduct any AMH study that does not have significant methodological weaknesses. As a result, the “true prevalence” and intensity of the negative effects associated with abortion can never be known with any great certainty. Noting this problem, the TFMHA review concurred with the view that the complexity of this field “raises the question of whether empirical science is capable of informing understanding of the mental health implications of and public policy related to abortion,” admitting that many research “questions cannot be definitively answered through empirical research because they are not pragmatically or ethically possible.”^4^

### Despite study limitations, statistically significant risks are regularly identified

While every observational study can be criticized for methodological weaknesses, it is also nonetheless true that is still possible to discover meaningful and actionable results. For example, research demonstrating elevated rates of mental health problems among women who feel pressured to abort contrary to their moral beliefs is generalizable to that specific subset of women. So while it is important to never generalize to *all women* who have abortions, insights can be gained from nearly any study when the results are properly narrowed to the limits of the population studied.^70^

**Figure 1.**
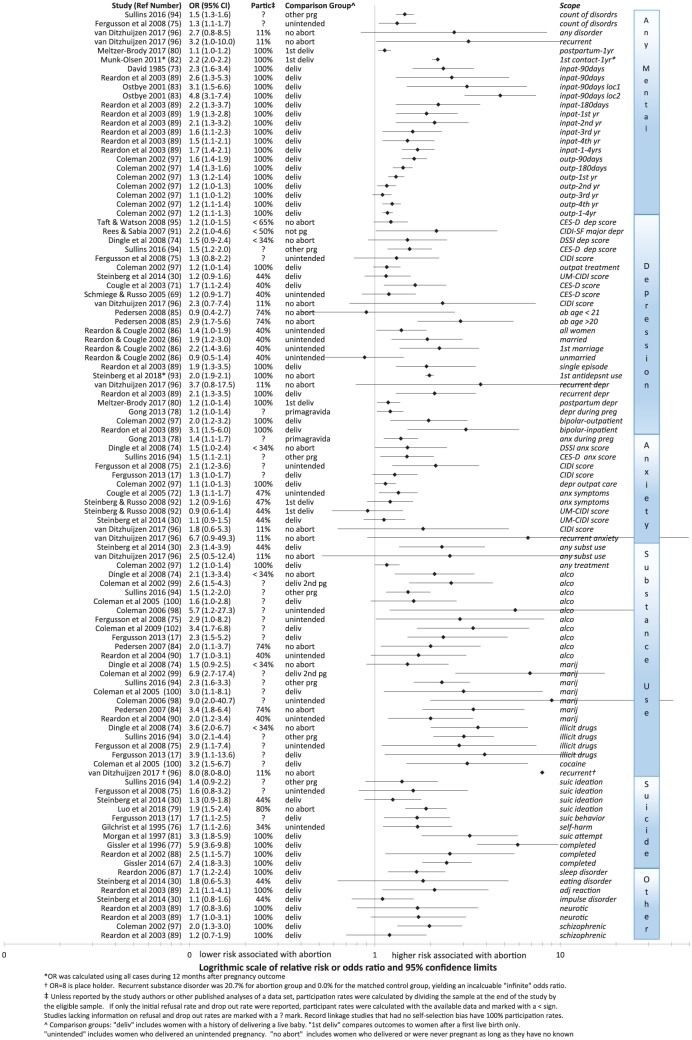
Relative risk of abortion relative to each study’s comparison groups.

Figure 1 shows the odds ratios (ORs) and 95% confidence interval (95% CI) for risks associated with abortion in all major studies published since 1995 organized by class of symptoms.^17,30,67,69,71–102^

While there are disagreements on how to best interpret these findings (to be discussed later), the findings themselves are not disputed. The results are organized into six sets: all classes of symptoms (segregated by inpatient and outpatient treatments when separately reported); depression and depression-related symptoms such as bipolar disorder; anxiety; substance use disorders (segregated by type of substance use when identified); and other disorders. Each row identifies the study reporting the results; the numeric relative risk (or OR) and CIs (also shown as a range in the forest plot); the participation rate of eligible women (after deducting refusals and dropouts) when identifiable; the group to whom the aborting women are being compared in the study; the forest plot; and an abbreviated description of the specific outcome, symptom, diagnostic scale, and/or time frame to which the statistic applies. Comparison groups include women carrying an unintended pregnancy to term, women delivering a child, women delivering a first pregnancy, women with no known history of abortion, women with any other pregnancy outcome other than abortion, and women not pregnant during the period studied.

What is most notable from Figure 1 is that the trend in results, including those reported by questionnaire and record linkage studies, is consistent. All but three odds ratios are above 1. In most cases, the lower 95% CI is also above 1, signifying statistical significance. Moreover, even among studies showing no significant difference (when the lower 95% CI is less than 1.0), the upper 95% CI is always above 1 and overlaps the statistically significant CIs of other studies.

This overlap is very important. For example, as can be seen in the depression grouping in Figure 1, the overlap of the 95% CIs in the findings of Schmiege & Russo 2005 and Cougle 2003 (both using different sampling rules for the same data set) demonstrates that *there is no actual contradiction in the findings* of these two studies. Whenever there is overlap in the CIs, this tells us that the variation in the respective relative risks reported by each study is within the expected range of variation given the limits of each study’s statistical power. Since findings only contradict each other when there is no overlap in the CIs, it is clear from Figure 1 that the minority of studies without statistically significant findings *do not contradict* the findings of studies with statistically significant findings. Claims to the contrary^69^ ignore the relevance of CIs and also the fact that studies with low statistical power are easily prone to Type II errors resulting in false negatives.

The risk of such false negatives is increased when there is also any risk of sample bias. In regard to abortion research, the risk of sample bias is especially high since questions about abortion are frequently associated with feelings of shame.^22,59^ The resulting selection bias due to self-censure and the high dropout rates of women at greatest risk of negative reactions also contributes to the misclassification of women concealing a history of abortion as non-aborters. In addition, some researchers choose to exclude groups such as women who abort wanted pregnancies,^69^ have later term abortions, or have other risk factors for more negative reactions (Table 1) and these methodological choices will also tend to shift results below statistical significance.

Despite these problems, the trend in findings, as shown in Figure 1, is very clear. Women who abort are at higher risk of many mental health problems.

This conclusion is strengthened by the variety of the study designs that have been conducted. Collectively, these studies examine a wide variety of different comparison groups, explore a diverse set of outcome variables, employ a large variety of control variables, and report on numerous outcomes over different time frames and/or at a variety of cross sections of time. Collectively, they reveal the following:

(a) There are no findings of mental health benefits associated with abortion. (These would be signified by the entire 95% confidence line being below 1.0.)
(b) The association between abortion and higher rates of anxiety, depression, substance use, traumatic symptoms, sleep disorders, and other negative outcomes is statistically significant in most analyses.
(c) The minority of analyses that do not show statistically significant higher rates of negative outcomes do not contradict those that do. (Shown by the upper bound of the 95% confidence overlapping the lower 95% CI of the statistically significant studies.)

A number of recent studies have also reported the population attributable risk (PAR) associated with abortion. This statistic estimates the percentage of an outcome that may be attributed to exposure to an abortion experience after statistically removing the effects associated with the available control variables.

**Figure 2.**
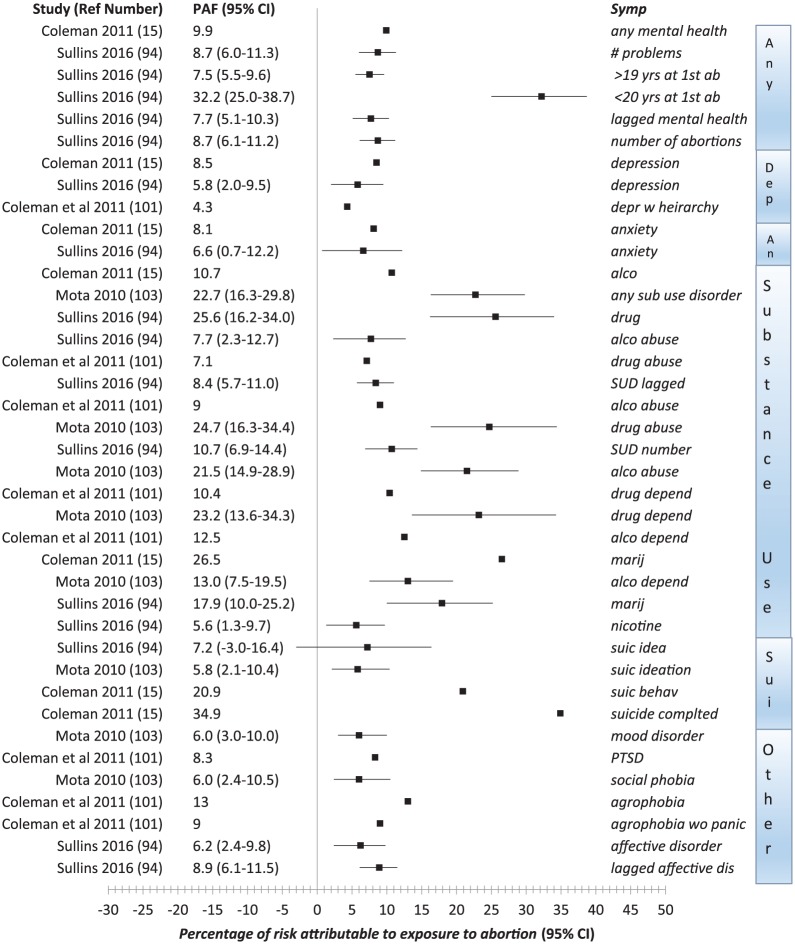
Population attributable fraction and 95% CI.

Fergusson was the first to report PARs identified in a prospective longitudinal cohort studied from birth to 30 years of age in New Zealand. He reported that the attributable risk ranged from 1.5% to 5.5%, but did not identify the PAR of specific mental health effects nor provide the CIs.^75^ Specific outcome PAR risks were also calculated by Coleman^15^ in her meta-analysis, but these were reported without CIs. These are shown in Figure 2 along with PAR estimates with 95% CIs that have been reported in three other studies.^94,101,103^

Of particular interest is a 2016 study by Sullins using the National Longitudinal Study of Adolescent to Adult Health that provided three models of analyses, including controls for 25 confounding factors. In addition, he conducted a fixed-effects regression analysis controlling for within-person variations to control “for all unobserved or unmeasured variance that may covary with abortion and/or mental health.”^94^ Sullins’ lagged models, employed as additional means of examining effects of prior mental illness, confirmed that the risks associated with abortion cannot be fully explained by prior mental disorders. He also identified a dose effect, with each exposure to abortion (up to the four) associated with a 23 percent (95% CI, 1.16–1.30) increased of relative risk of subsequent mental disorders.

Collectively, the findings shown in Figure 2 suggest that substance use disorders appear to be most strongly attributable to abortion. Put another way, assessments of substance use (perhaps indicating self-medicating behavior) may be one of the more sensitive measures of difficulties adjusting to post-abortion.^96^ Conversely, at least some research has shown that other outcomes, such as variations in self-esteem, may be unaffected, or only weakly associated with abortion.^38^ Alternatively, some outcomes may appear to be less strongly associated with abortion because women are receiving successful treatment, such as medication for depression or anxiety, that would obviously suppress these associations with abortion.

### Prior mental health and co-occurring factors explain at least part of the effects

As shown in Table 1, a history of mental health problems is a risk factor for higher rates of mental health problems following abortion as compared to women without a history of mental health problems. This association has been known since at least 1973 when a case series identified several pre-existing mental health factors that could be used to identify the women who were most likely to experience subsequent psychopathology.^32^ The authors of that study recommended that a low-cost computer scored Minnesota Multiphasic Personality Inventory assessment could effectively identify women who could benefit from additional pre- and post-abortion counseling.

Both AMH proponents and AMH minimalists agree that prior health is a major factor in explaining the negative reactions observed post-abortion. There are differences, however, in how proponents and minimalists distinguish, interpret, and emphasize the interactions between prior mental health, the abortion experience, and subsequent mental health.

AMH proponents see poor prior mental health as contributing to the risk that a woman (a) may become pregnant in problematic circumstances; (b) may be more vulnerable to pressure or manipulation to have an abortion contrary to personal preference, maternal desires, or moral ideals; and (c) may have fewer or weakened coping skills with which to process post-abortion stresses. In addition, from the perspective of abortion as a potential stressor, women exposed to prior traumatic experiences may be more predisposed to experiencing abortion as another traumatic experience.

In contrast, AMH minimalists tend to interpret the evidence that a high percentage of women having abortions have prior mental health issues as the primary explanation for higher rates of mental illness observed after abortion.^5,7,104,105^ From this perspective, women with mental health problems are more likely to engage in risk-taking behavior and to experience more problematic pregnancies and are more likely to choose abortion. It is also hypothesized that pregnant women with pre-existing mental health problems may be more inclined to choose abortion because they recognize that they are likely to fare worse if they deliver and try to raise an unplanned child.^106,107^ The higher rates of mental health issues following abortion, therefore, may be mostly explained as just a continuation of pre-existing mental health problems rather than a direct and independent cause of mental illness. While a few minimalists suggest that the underlying cause of mental health problems observed after abortion can be entirely explained by prior mental health defects or co-occurring stressors,^30,82^ I have been unable to find any researchers who have denied that abortion can *contribute* to mental health problems.

A closely related issue is that a history of being physically and/or sexually abused is a co-occurring risk factor for both mental health problems and abortion.^92,94,108–110^ Obviously, both sides agree that trauma from prior abuse can harm mental health. Also, at least from the clinical perspective of AMH proponents treating women with a history of both abortion and abuse, a history of abuse may increase the vulnerability of women consenting to unwanted abortions.

The differences between AMH minimalists and proponents on these issues will be more thoroughly discussed later. At this point, it is sufficient to note that both sides agree that poor prior mental health is a major predictor of higher rates of mental health problems after an abortion. Moreover, both sides agree that there should be mental health screening of women seeking abortion^24–30,32–38,58^ precisely because the “abortion care setting may be an important intervention point for mental health screening and referrals”^30^ due to the higher concentration of women with previous and subsequent mental health issues. At the very least, a history of abortion is a useful marker for identifying women at greater risk of mental health problems and a corresponding elevated risk of a variety of related chronic illnesses^111^ and reduced longevity.^112,113^

### A summary of agreements with difference in emphasis

Table 2 summarizes specific factual propositions to which the vast majority of both AMH minimalists and AMH proponents would agree. As indicated in the table, each side may typically emphasize some points over others and might underemphasize, reluctantly admit, or even evade discussion of some of these propositions. Still, while some may quibble over the exact formulation of any particular proposition in Table 2, the underlying consensus relative to each proposition is easily discernible in the body of references by both sides cited in this review.

**Table 2.** Variations in emphasis on conclusions generally shared by AMH minimalists and AMH proponents.

Propositions regarding agreed upon facts	AMH minimalists	AMH proponents
Abortion contributes to mental health problems in some women.	Admits	Emphasizes
The majority of women do not have mental illness following abortion.	Emphasizes	Admits
A significant minority of women do have mental illness following abortion.	Admits	Emphasizes
Risk factors exist that identify women at higher risk.	Admits	Emphasizes
The observed higher rates of mental illness in women with a history of abortion may be partially or mostly attributable to common risk factors.	Emphasizes	Admits
There is insufficient evidence to prove that abortion is the sole cause of the higher rates of mental illness associated with abortion.	Emphasizes	Admits
There is substantial evidence that abortion contributes to the onset, intensity, and/or duration of mental illness.	Admits	Emphasizes
A substantial number of women attribute their mental health problems, at least in part, to their abortion experiences.	Admits	Emphasizes
There is no evidence that abortion can resolve or improve mental health.	Admits	Emphasizes
A history of abortion can be used to identify women at higher risk of mental health issues who may benefit from referrals for additional counseling.	Admits	Emphasizes
There is a dose effect, wherein exposure to multiple abortions is associated with higher rates of mental health problems.	Admits	Emphasizes
No single study design can adequately address and control for and address all the complex issues that may related to the AMH issues.	Emphasizes	Emphasizes

AMH: abortion and mental health.

In summary, the consensus of expert opinion, including that of both AMH proponents and minimalists, is that (a) a history of abortion is consistently associated with elevated rates of mental illness compared to women without a history of abortion; (b) the abortion experience can directly *contribute* to mental health problems in some women; (c) there are risk factors, including pre-existing vulnerability to mental illness, which can be used to identify the women who are at greatest risk of mental health problems following an abortion; and (d) it is impossible to conduct research in this field in a manner that can definitively identify the extent of any mental illnesses following abortion, much less than the proportion of disorders that can be reliably attributed solely to abortion itself.

## Obstacles in the way of research, understanding, and consensus

Facts are facts. But there is plenty of room for disagreement regarding which facts are generalizable, much less on how to best synthesize and interpret sets of facts, especially when there are flaws in the research and gaps in what one would want to know. Indeed, the greater the ideological differences between people regarding any question, the easier it is to disagree about what the available evidence really means. As shown in Table 2, even areas around which there is a fundamental agreement by experts under sworn testimony may appear muddied by shifts of emphasis and the insertion of nuances that may be technically true but misleading to non-experts who imagine there are simple, global answers.

For example, the same APA task force which produced the list of risk factors shown in Table 1 did not highlight these findings in their press releases with a recommendation for screening. Instead, the centerpiece of their press release^114^ was the report’s conclusion that “the relative risk of mental health problems among *adult* women who have a *single*, legal, *first-trimester* abortion of an *unwanted* pregnancy for *nontherapeutic* reasons is no greater than the risk among women who deliver an *unwanted* pregnancy”^7^ (italics added).

This statement was widely reported as the APA officially concluding that abortion has no mental health risks. But as shown in Table 1, this reassuring conclusion was actually couched in nuances which make it applicable to only a minority of women undergoing abortions on any given day. It excludes the 48%–52% of women who already have a history of one or more abortions,^64^ the 18% of abortion patients who are minors,^115^ the 11% of patients beyond the first trimester,^116^ the 7% aborting for therapeutic reasons regarding their own health or concerns about the health of the fetus,^117^ and the 11%–64% whose pregnancies are wanted, were planned, or for which women developed an attachment despite their problematic circumstances.^38,50,51^

The above example demonstrates that the same set of facts, presented and interpreted by AMH minimalists in a way that suggests that *few women* face any risk of negative reactions to abortion, could also have been worded by AMH proponents in a way that would have underscored a conclusion that *most women* having abortions are at greater risk compared to the minority who have no risk factors.

This points to one of the greatest hindrances in the advance of knowledge: the tendency to use nuances to dodge direct engagement with the ideas, evidence, and arguments which threaten one’s own preconceptions.

Therefore, one of the purposes of the following discussion is to invite direct engagement and thoughtful responses to the specific obstacles identified below.

### Intrinsic biases in the assessment of evidence are nearly impossible to avoid

Everyone, even the most “objective” scholar, has developed shortcuts in their thinking and beliefs. These shortcuts (or biases) help us to (a) be more efficient in drawing conclusions and making decisions and also (b) be more consistent in how we perceive ourselves and reality, or conversely, to avoid the stress of cognitive dissonance which occurs when some fact or experience clashes with our core beliefs and values.

Our biases are not just personal. They also have a communal element. We tend to adopt the biases of our peers for several practical reasons. First, by adopting the opinion of our peers as our own, we are embracing a collective wisdom that frees us from the need to deeply research and consider every idea on our own. Second, the more completely our beliefs are aligned within our community of peers, the less we will face conflict and suspicion. Obviously, there is never perfect alignment or cessation of independent thinking. But the tendency to accept the “conventional truths” of one’s peers as “fact” is a very real phenomenon.

The impact of biases among academics on the interpretation of data and suppression of contrary opinions has been well documented.^118–123^ For example, identical studies, for which the results are the only difference, are more likely to be lauded or condemned^122–125^ by peer reviewers when the results confirm or conflict with the reviewer’s own biases. In the fields of psychology and psychiatry, such confirmatory bias may contribute to the promotion or suppression of research findings that favor liberal causes.^125–128^ In one study, only one-fourth of reviewers noted a major methodological problem in a fake study that agreed with their preconceptions, while 72% quickly raised an objection about the problem when presented with a nearly identical fake study in which the results challenged their preconceptions.^123^ The only way to eliminate result-based bias, the author suggests, would be to solicit reviews only on the relevance of a study’s methodology, withholding the actual results and discussion of results, since the latter are the actual drivers of confirmatory bias.^123^

While much of the confirmatory bias observed in peer reviewers may be unconscious,^129^ at least one survey of 800 research psychologists found high rates of admissions that they or their colleagues would openly and knowingly discriminate against conservative views when providing peer review (34.2%), awarding grants (37.9%), or making hiring decisions (44.1%).^130^ The authors noted that this admission of conscious ideological bias was likely just the tip of the iceberg compared to confirmatory bias since “[i]t is easier to detect bias in materials that oppose one’s beliefs than in material that supports it.^124^ Work that supports liberal politics may thus seem unremarkable, whereas work that supports conservatism is seen as improperly ideological.”^130^

In addition to blocking publication of good research, ideological and confirmatory bias may also contribute to poorly designed studies and/or carelessly interpreted findings that advance a preferred viewpoint.^118,126,131–133^

Social psychologist Jonathan Haidt, a self-proclaimed liberal specializing in the foundations of morality and ideology, has argued that that the vast majority of psychologists are united by the “sacred values” of a “tribal-moral community” which is politically aligned with the liberal left.^134^ This shared moral superiority,^129^ he says, both “binds and blinds” their community.^134^ The risk of “blindness” occurs because the lack of sufficient political diversity predisposes the community of psychologists to “embrace science whenever it supports their sacred values, but they’ll ditch it or distort it as soon as it threatens a sacred value.”^134^

In regard to the abortion, mental health controversy, studies by AMH minimalists tend to be written in a way that minimizes any disruption of the core pro-choice aspiration that abortion is a civil right that advances the welfare of women.^135^ The research on confirmatory bias discussed above, therefore, suggests that studies by AMH proponents are more likely to be unfavorably reviewed and rejected.^136^

An excellent example of this result-based bias was the four rejections reported by David Fergusson, former director of the Christchurch Health and Development Study, which followed 1265 children born in Christchurch, New Zealand, for over 30 years.^137^ Fergusson, a self-proclaimed pro-choice atheist, believed that his data would help to prove that AMH proponents were wrong.^137^ But when he ran his analyses, he found that even after controlling for numerous factors, abortion was indeed independently associated with a two-to threefold increased risk of depression, anxiety, suicidal behaviors, and substance abuse disorders.^17,138^ Though his findings were opposite to his preconceptions, he submitted them for pubication anyway. It was then that he ran into a wall of ideologically driven rejections and was even asked by the New Zealand government’s Abortion Supervisory Committee to withhold the results.^137^

Similarly, Ann Speckhard,^139^ another pro-choice AMH proponent and an associate professor of psychiatry at Georgetown University Medical School, has complained,

Politics have also stood in the way of good research being conducted to examine psychological responses in a nationally representative sample to all pregnancy outcomes: live birth, miscarriage, induced abortion, and stillbirth (and perhaps even including adoption). I offered in 1987 to our National Center for Health Statistics a simple mechanism for collecting such data via a short interview to be attached to an already existing survey—but fear of the answers—on both sides of the issue staunchly squelched the idea.

The problem is that even trained scientists struggle with being purely objective—especially regarding issues that may touch one’s own core beliefs, values, and experiences. What makes Fergusson’s experience particularly unique is that he chose to publish his findings even though they contradicted his own worldview. How many other researchers, expecting to prove mental health benefits from abortion but finding the opposite, might be tempted to withhold their findings, or worse, to redesign their study in ways that would obfuscate their results in order to declare that a lack of statistically significant results “proved” that there was no need to look further? This concern is heightened by the refusal of AMH minimalists to allow examination of their data by AMH proponents,^140^ as will be discussed in more detail later.

Just as lawyers are taught to never ask a question at trial to which you do not already know the answer, researchers engaged in any field where there are “adversarial” positions may often be hesitant to cooperate in a mutual pursuit of objective truth.^141^ This fear of admitting the validity of “the other side’s” concerns is also reflected in the admission by pro-choice feminists that they are afraid to publicize the existence of their own post-abortion counseling programs.^44,142^

These concerns regarding bias surrounding AMH issues are further heightened by the fact that many professional organizations, including the APA, have taken official political positions defending abortion as a “civil right.”^135^ In defense of that political position, Nancy Russo, a member of the APA’s TFMHA, has stated that “whether or not an abortion creates psychological difficulties is not relevant”^143^ and has been a proponent of the APA taking a pro-active role in aggressively attacking the credibility of studies by AMH proponents.^144^ The problem with professional organizations taking a political position on abortion is that any subsequent acknowledgment of negative mental health effects linked to abortion might then embarrass the APA, and/or other professional organizations that have committed themselves to the agenda of defending abortion as a civil right, and thereby creates an ideological obstacle in objectively evaluating new evidence.

### There are different rates of exposure to the highest risk and lowest risk archetypes

This leads us to an important and perhaps closely related observation. It is not only political, philosophical, or ideological beliefs that contribute to the AMH controversy. Conflicts in the perceiving AMH controversy are also colored by direct and indirect *personal experiences*. The fact that pro-choice feminists are more focused on feelings of relief and other liberating aspects of having a right to abortion^3^ may be accurately representing their own positive personal experiences. Conversely, anti-abortion conservatives, who presume that AMH problems are common, may be accurately representing their own relative rate of exposure to negative experiences.^3^

Support for this hypothesis is found in a study based on structured interviews of women following their abortions conducted by Mary Zimmerman^48^ in which she found that approximately half of the women she interviewed could be classified as “affiliated” (more goal oriented, more educated, less dependent on the approval of others, and more likely to abort for their own self-interest) and the other half as “disaffiliated” (less career oriented, less educated, more dependent on the approval of others, and more likely to abort to please others). When she interviewed her sample 6 weeks after their abortions, Zimmerman^48^ found that only 26% of “affiliated” women were struggling with “troubled thoughts” about their abortions compared to 74% of “disaffiliated” women, a threefold increase. A similar disparity relative to personality types was observed by Major et al.^145^

It is reasonable to assume that friends and associates of highly educated research psychologists are more likely to be skewed toward the “affiliated” than the “disaffiliated.” If so, the personal experience of such AMH skeptics may be dominated by the observation that they and their closest friends have generally coped well with any exposure to abortions.

Conversely, AMH proponents, especially those who directly meet and counsel women having problems dealing with past abortion^45^ may have little or no experience with women who have had positive abortion experiences. The concentrated experience of meeting with scores or hundreds of women struggling with past abortions would understandably incline AMH proponents to believe that negative experiences with abortion are more common than positive ones.^146^

In short, applying the general rule that people (including scientists) tend to look for and believe data that confirm their preconceptions, and are disproportionately skeptical of data that conflict with their preconceptions, both AMH skeptics and AMH proponents are at risk of preferentially interpreting their personal exposure to abortion’s risks and benefits as applicable to the general population.

While women having abortions will fall across the entire spectrum of risk factors, it is useful for this review to consider two hypothetical women at opposite ends of any risk-benefits analysis: (a) “Allie All-Risks,” the worst possible candidate for an abortion and (b) “Betsy Best-Case,” with no known risk factors:

- “Allie All-Risks” is 15 years old. A victim of verbal, emotional, and physical abuse, including three incidents of sexual molestation, she has low self-esteem with bouts of anxiety, depression, and suicidal ideation. While her parents are not regular churchgoers, she attended a Catholic grade school, believes in God, and believes abortion is the killing of a baby. She is not a good student and has no concrete career goals. She has always wanted to be a mother, loves babies, and fantasizes about how she will find fulfillment in giving the love to her children that she never received from her own mother. Given Allie’s yearnings for escape, acceptance, and true love, she is vulnerable to the seductions of a 22-year-old womanizer with whom she falls madly in love and aspires to a happy future. When she learns she is pregnant, her initial reaction is excitement. While not planned, the pregnancy is welcomed. She believes she can now start building a family with her lover. But this fantasy is immediately crushed when he tells her that they can’t afford it, that neither of them are ready for it, and that if she decides to continue the pregnancy, he will leave her. She feels she has no choice. She can’t imagine losing him. In addition, her parents would be furious and insist on an abortion, too. Allie’s initial excitement at being pregnant is replaced by despair. Indeed, given her need to please others, she gives in with barely a complaint. Her mild protests about “their choice” go unnoticed. The day of the abortion she whispers: “Good bye. I don’t want to do this to you. But I don’t have a choice.” Immediately after the abortion, Allie feels a mild relief that the dreaded procedure is now behind her and hopes her boyfriend will be content, but alongside that relief are feelings of emptiness and loss that seem to grow stronger with every passing week. She begins to have obsessive thoughts. Her baby is no longer in her body, but it is constantly in her thoughts.
- “Betsy Best-Case” is 32 years old. She has no history of mental illness and has a good family life. Her parents were both well-educated secularists. They preach education, hard work, and honest success as the only ethical standards Betsy needs to guide her. Betsy is popular, has many friends, and has always had high career aspirations, toward which, with grit, she has proudly made good progress. Even as a child, Betsy had little or no interest in being a mother. Married to her career, she now has even less interest in maternity. Having successfully used birth control since she was 15, when her mother got her an IUD, Betsy is shocked when she realizes she is pregnant. But contraceptive failures happen. Her decision to abort is immediate and made without any emotional conflict. When she flips through the state mandated informed consent booklet given to her at the abortion clinic, the pictures of developing fetuses have no effect. Betsy has seen similar photos many times in the past. She has a strong philosophical belief, based on years of engagement in minor abortion debates, that the value of being a “person” is not based on biological features but rather on the development of a psychological, purpose-filled, self-actualized human being far beyond anything to which a 9-week-old fetus could yet lay claim. Betsy is not surprised when her abortion is completed without drama or even a tinge of angst. She thinks of it rarely. The only negative feelings ever associated with it come when she hears the right of women to choose abortion attacked by self-righteous busybodies who should know better.

Hopefully, any reader can see and respect that the Allie and Betsy’s abortion experiences are very different. One is focused on her loss and the other on how her abortion helped her to avoid any loss. Given these differences, it would be unfair to them try to interpret their abortion experiences from within a single ideological framework. Similarly, the women who reside at different places along the wide spectrum between the extreme poles of Allie and Betsy are also very different and unique.

We will employ Allie and Betsy in our discussion later in this review. But for now, let them simply stand as examples of why AMH skeptics may, from personal experience, presume that Betsy is “typical” of abortion patients, while AMH proponents may presume that Allie is more “typical.” This difference in regard to how each side of the AMH controversy views the “typical” abortion patient is likely to impact how they interpret AMH research in their efforts to describe the experience of “most” women.

### There are multiple pathways for AMH risks

Despite the convenience of standard diagnostic criteria, mental illnesses do not necessarily fit into neat, single classifications with distinct and exclusive symptoms arising from a single cause for each illness.^147^ As noted in one review of the psychiatric complications of abortion,

A psychiatric complication is a disturbance that occurs as an outcome that is precipitated or at least favored by a previous event …. *Every psychiatric outcome is of a multi-factorial origin.* Predisposing factors including polygenic influence and precipitating factors such as stressful events are involved in this outcome; in addition, there are modulating, both risk and protective, factors. The impact of the events depends on how they are perceived, on psychological defense mechanisms put into action (unconscious to a great extent) and on the coping style.^18^ (Emphasis added)

An abortion does not occur in isolation from interrelated personal, familial, and social conditions that influence the experience of becoming pregnant, the reaction to discovery of the pregnancy, and the abortion decision. These factors will also affect women’s post-abortion adjustments, including adjusting to the memory of the abortion itself, potential changes in relationships associated with the abortion, and whether this experience can be shared or must be kept secret. These are all parts of the abortion experience. Therefore, the mental health effects of abortion cannot be properly limited to the day on which the surgical or medical abortion takes place. The entirety of the abortion experience, including the weeks before and after it, must be considered.

Moreover, there is no reason to believe that there is a single model for understanding, much less predicting, all of the psychological reactions to the abortion experience. Miller alone identified and tested six models for interpreting psychological responses to abortion and concluded that

theoretical approaches that emphasize unitary affective responses to abortion, such as feelings of shame or guilt, loss or depression, and relief may be missing an important broader picture. To some extent what appears to happen following abortion involves not so much a unitary as a broad, multidimensional affective response.^148^

The APA’s TFMHA proposed four models: (a) abortion as a traumatic experience, (b) abortion within a stress and coping perspective, (c) abortion within a socio-cultural context, and (d) abortion as associated with co-occurring risk factors.^7^ Additional models could be built on biological responses,^149,150^ attachment theory,^151–154^ bereavement,^153,155–158^ complicated, prolonged or impacted grief,^159–163^ambiguous loss,^156,161,164–167^ or within a paradigm of psychological responses to miscarriage.^74,168–170^

The complexity of considering so many models, or pathways, combined with the multiplicity of symptoms women attribute to their abortions,^45^ contributes to discord in the literature produced by AMH proponents and AMH minimalists.

When there is no agreement on what outcomes are relevant or what theoretical pathways should be investigated, there are countless reasons to disagree about both (a) the adequacy of any specific studies and (b) how any specific set of findings should be best interpreted.

### Women may simultaneously experience both positive and negative reactions

The act of undergoing an abortion can be both a stress reliever and a stress inducer.^171^ It may relieve one’s immediate pressures and concerns while also leaving behind issues that may require attention immediately or at a future date. Positive and negative feelings can co-exist and frequently do.^38,39,48,50,166,172^

In one study,

Almost one-half also had parallel feelings of guilt, as they regarded the abortion as a violation of their ethical values. The majority of the sample expressed relief while simultaneously experiencing the termination of the pregnancy as a loss coupled with feelings of grief/emptiness.^166^

Another study found that 56% of women chose both positive and negative words to describe their upcoming abortion, 33% chose only negative words, and only 11% chose only positive words.^62^ The women at greatest risk of experiencing negative reactions immediately and in the short term following an abortion are those who feel most conflicted about the decision to abort or have other pre-existing risk factors.^39,45,82,173^

Applying this insight to our polar extremes, Annie All-Risks would be more likely to experience strong negative feelings more profoundly than her feelings of relief, whereas Betsy Best-Case would be more likely to focus on her relief than any doubts or reservations. Moreover, because Annie has low expectations for coping well (itself a TFMHA risk factor), she may be less likely to agree to participate in a follow-up study. The faster she can get out of the abortion clinic without talking to anyone, the better. Conversely, Betsy is confident that her decision is right and will improve her life and is therefore much more likely to participate.

### What “most women” experience cannot be reliably measured

As will be further discussed later, the fact that positive and negative feelings can co-exist makes it difficult, and potentially misleading, to describe any single reaction to abortion as the “most common,” given the fact that (a) it is very rare for women to have a single reaction and (b) typically, over half of women asked to participate surveys regarding their abortion experiences refuse or drop out. Obviously, it is impossible to know what the most common reaction of women is based on surveys of only a minority of self-selected women.

This insight also underscores the difficulty of making any generalizations regarding prevalence rates from any study involving volunteer participation or questionnaires. Broadly speaking, there are three groups of women: (a) those with no regrets or negative feelings, (b) those with deep regrets and profound negative feelings, and (c) those with a mix of feelings, including contradictory feelings. As discussed above, the best evidence indicates that women with the most negative feelings are least likely to agree to participate in studies initiated at abortion clinics. But it also follows that women with no regrets are unlikely to be represented in studies of women seeking post-abortion counseling. Both of these factors underscore that it is impossible to accurately measure how “most” women react to their abortion experience when participation in research is voluntary.

### The degree of reactions can widely vary and there is no reasonable cutoff for concern

Not all negative emotions constitute a diagnosable mental illness. Therefore, the fact that only a minority of women have diagnosable mental illnesses following abortion does not preclude the possibility that a majority experience negative emotional reactions.

Structured interviews of women who received abortions at participating clinics reveal that the majority report at least one negative emotion that they attribute to their abortions.^48,172^ Given the relatively high rate of women refusing to participate in these follow-up studies, it is likely that the actual percentage of women having at least some negative reactions is well over half.^174^ Similarly, retrospective questionnaires of women also reveal that over half attribute at least some negative reactions to their abortions.^50^

The opinion that negative reactions are experienced by the majority of abortion patients is also shared by a number of abortion providers, such as Poppemna and Henderson:^175^

Sorrow, quite apart from the sense of shame, is exhibited in some way by virtually every woman for whom I’ve performed an abortion, and that’s 20,000 as of 1995. The sorrow is revealed by the fact that most women cry at some point during the experience …. The grieving process may last from several days to several years.

Similarly, Julius Fogel, who as both a psychiatrist and OB-GYN and as a pioneer of abortion rights performed tens of thousands of abortion, testified that while abortion may be necessary and generally beneficial, it always exacts a psychological price:

Every woman—whatever her age, background or sexuality—has a trauma at destroying a pregnancy. A level of humanness is touched. This is a part of her own life. When she destroys a pregnancy, she is destroying herself. There is no way it can be innocuous. One is dealing with the life force. It is totally beside the point whether or not you think a life is there. You cannot deny that something is being created and that this creation is physically happening …

Often the trauma may sink into the unconscious and never surface in the woman’s lifetime. But it is not as harmless and casual an event as many in the pro-abortion crowd insist. A psychological price is paid. It may be alienation; it may be a pushing away from human warmth, perhaps a hardening of the maternal instinct. Something happens on the deeper levels of a woman’s consciousness when she destroys a pregnancy. I know that as a psychiatrist.^176,177^

This distinction between negative reactions and diagnosable mental illness is another important reason why AMH proponents and minimalists *appear* to disagree more than they really do. When AMH proponents make statements about “most women” which imply that negative reactions are common, they are including women who attribute any negative reactions to their abortions even if the reactions fall short of fitting a standard diagnosable illness.^45^ Conversely, when AMH minimalists insist that “most women” do not experience mental illness due to their abortions, they are excluding the women who have negative feelings, even if unresolved and disturbing, on the grounds that (a) the symptoms do not rise above the threshold necessary to diagnose a clinically significant mental illness and (b) the symptoms cannot be strictly attributed to the abortion experience alone.^7^

In short, if pressed, both sides would agree that the best evidence indicates that most women do experience at least some *negative feelings* related to their abortion experiences. Yet at the same time, the majority do not experience *mental illnesses* (as defined by standard diagnostic criteria) that can be *solely attributed* to their abortions.

This brings us to a more general problem regarding the claim that “the majority” of women experiencing relief following their abortions.^178,179^ For women who do have strong negative feelings, such global denials of their personal experience may be demeaning. Even if these women’s negative reactions fall short of being classified as mental illnesses, it is reasonable for them to take offense at the AMH minimalist’s assertion that abortion does not involve any emotional risks, much less that the only women troubled by abortion are those who already had prior emotional problems.^180^ In short, publicity suggesting that abortion has no psychological effects may have the unintended effect of making women who do struggle with a past abortion feel like “freaks” who are unable to handle their abortions as easily as “everyone else.”^45^

Even if it could be proven that 99% of women who had abortions experienced more benefit than harm, that would still not justify ignoring the 1% who experienced more harm than good. Majorities matter in elections. But in regard to medical ethics and public policy, negative reactions are important among even a minority of patients … especially when it is possible to screen for risk factors that identify the patients at greatest risk of adverse reactions.

### Negative reactions may manifest themselves over a very long time frame

Most studies can only capture evidence spanning very limited timeframes. In the 1960s and 1970s, most studies of emotional reactions after abortion were based on volunteer samples limited to a few hours, days, or weeks after the abortion. These studies typically found negative outcomes in the range of 10%–20% of their volunteer samples. Early reactions, however, are not necessarily predictive of longer range reactions.^38^ Subsequent studies revealed that the percentage of women experiencing negative reactions increases with time, along with a significant drop in decision satisfaction and feelings of relief.^39,148^

For example, in a study led by TFMHA chair Brenda Major, volunteers interviewed at an abortion clinic reported a significant decline in their Brief Symptom Inventory Depression scores 1–2 h after their abortions (T2, 62% decline) compared to their scores an hour before their abortions (T1, asking women to rate their depression for the month prior to the abortion). But at the 1-month follow-up (T3), depression scores rose 91% above their post-abortion (T2) score and continued to get higher, up to 118% at the 2-year follow-up (T4).^39^ Notably, this study had a 30% dropout at the 1-month follow-up (T3) and a 50% dropout at the 2-year follow-up (T4). In addition, the self-selection bias of this volunteer sample was further magnified by the study protocol that also excluded women aborting an intended pregnancy or a second trimester pregnancy, two of the risk categories for elevated risk of negative reactions.

The fact that negative reactions may unfold over a long period of time is also evident from retrospective surveys. For example, one survey of women seeking post-abortion counseling found that only 24% claimed they had always been aware of negative feelings regarding their abortions. Of the remainder, less than half reported “doubts or negative feelings” within the first 3 years, while 100% were experiencing negative feelings by the time they sought post-abortion counseling.^45^ A similar survey found that 70% of women seeking post-abortion counseling reported that there had been a time after their abortions when they would have denied having any negative feelings.^181^ The first appearance of negative emotions may occur even as late as menopause.^182^

It is likely that there are patterns relative to which women are at greater risk of experiencing early negative reactions and those who are likely to experience later reactions. Zimmerman, for example, found that 74% of “disaffiliated” women were struggling with negative thoughts about their abortions, three times the rate reported by “affiliated” women.^48^ Thus, it is easy to predict that our archetype Annie All-Risks would likely be among those who would have immediate negative reactions. After all, she felt coerced into aborting an unplanned but welcomed pregnancy against her maternal preferences and moral beliefs. In addition, given her history of abuse and psychological problems, her coping skills were already stretched to the limit prior to her abortion.

Similarly, it is also easy to imagine that Betsy Best-Case would cope well in the immediate hours, days, months, and even years after her abortion. She freely chose to abort a pregnancy that was both unintended and unwanted for rational reasons. She also had strong coping skills and could easily compartmentalize any “socially induced” doubts into the “deeper levels” of her consciousness.

Clinical experience indicates, however, that there is no certainty that Betsy will *always* remain symptom free. Subsequent reproductive events such as miscarriage, infertility, or even a wanted birth may unexpectedly trigger existential crises deeply intertwined with a nearly forgotten abortion experience.^24,37,40,45^ Similarly, life events that trigger introspection such as the death of a loved one, or a later religious conversion, may trigger a redefinition of past choices and experiences in a way that may include obsessive guilt and self-condemnation.^45^ An example of a “perfect decision” being reinterpreted as a woman’s worst decision is found in this posting at a post-abortion counseling site:

I had an abortion when I was 22 years old. Now it is haunting me. I think about it every day of my life. I have so much regret. I wish I could turn the clock and undo my mistakes. I am not coping. The guilt is too much. At that time the decision was perfect. But now it kills me day by day. Please help me. I don’t trust anyone with this secret.

AMH minimalists might reasonably argue that it is the subsequent trigger, the miscarriage, or religious conversion, that is the “true cause” of later distress. But efforts to apportion blame for the “true cause” of distress over a prior abortion simply disrespects the real experience of women who seek, desire, or need post-abortion counseling. Whatever the trigger, whatever the contributing factors, the internal turmoil over a past abortion is centered on, or at least intertwines with, the abortion and will not be resolved by pretending the abortion is not part of the problem.

Based on reports of clinical experience, we would hypothesize that delayed reactions are most frequently triggered by (a) subsequent reproductive experiences, including reproductive difficulties and (b) experiences that lead to introspection and reevaluation of one’s overall life course or moral integrity.^45^ Conversely, the more risk factors that are present, especially feelings of coercion and attachment combined with weakened coping skills, are predictive of more immediate negative reactions.

The great variability in the time frame for negative reactions greatly complicates the interpretation of studies examining limited time frames, and even those covering long time frames but at infrequent intervals. For example, two studies examined Center for Epidemiological Studies depression scores (CES-D) collected by the National Longitudinal Study of Youth (NLSY) an average of 8 years after an abortion.^69,86^ But the NLSY was not designed to study reproductive or mental health and had a very high concealment rate regarding past abortions. Moreover, the single year in which depression was evaluated in the NLSY could only provide a bit of cross-sectional information about the women surveyed. While the passage of time may have helped to identify some delayed reactions, it would also miss cases where women have gone through a healing or recovery process during the 8 years (on average) for which there was no data. Moreover, the NLSY’s single measure for current depression, the CES-D, did not account for women who were being successfully treated for depression with medication.

In short, questionnaires which lack abortion-specific retrospective questions such as “Did you *ever* experience significant negative feelings about a past abortion?” followed by questions regarding the timeline for each type of mental health outcome being studied^45,50,183^ are simply capturing cross-sectional data. Cross-sectional data regarding current symptoms will simply miss symptoms that have ceased, either due to medication, counseling, or by the healing effects of time or a replacement pregnancy. It will also miss symptoms that may be delayed beyond the date of the assessment. As a result, data from general prospective studies like the NLSY simply cannot tell us anything about the “true prevalence rate” of depression associated with abortion.

The weakness of such general purpose prospective studies also explains why AMH proponents and AMH minimalists can look at the same data and come to different conclusions. For example, the first analysis of NLSY CES-D scores relative to women with a history of abortion found that depression was highest among married women with a history of abortion (OR = 1.92; 95% CI = 1.24–2.97) and among women in their first marriage in particular (OR = 2.23; 95% CI = 1.36–3.74).^184^ Since CES-D scores did not significantly vary among unmarried women, the combined results for all women (OR = 1.39; 95% CI = 1.02–1.90) were barely significant.^184^ The significance of marital status may indicate that abortion-related depression after an average of 8 years may be triggered by subsequent pregnancies in marriage. In any event, given the weakness of this data set, it was a trivial matter for AMH minimalists^69^ to use different selection criteria, excluding a subgroup of women at greatest risk of negative reactions to abortion, in order to shift the lower 95% CI for all women below 1 (OR = 1.19; 95% CI: 0.85–1.66) in their reanalysis of the NLSY data. Notably, their analysis also excluded results segregated by marital status, the finding most significant in the earlier study. Based on these weaknesses, it was simply misleading for Schmiege and Russo^69^ to interpret their reanalysis as conclusive evidence that abortion does not contribute to the risk of depression in some women. Their overreaching conclusions were particularly unjustified in light of the fact that the NLSY data set was also tainted with a 60% concealment rate regarding past abortions^185^ and the CES-D scale inquired about only depression in the prior week and was administered in only once, an average of 8 years after the abortions.

In summary, the efforts to estimate the prevalence rate of negative reactions to abortion are complicated by (a) the wide variety of reactions, (b) the existence of both early and delayed reactions, (c) a wide variety of triggers for delayed reactions, and (d) the prospect that in any assessment years after the abortion, a number of women who previously had significant reactions may have experienced full or partial recovery by the time of that assessment. Each of these factors would tend to skew the results of any prevalence estimates based on questionnaires toward underestimating the total lifetime risks.

### Self-censure and defense mechanisms contribute to underreporting of sequelae

Data collected to investigate reactions to abortion may also be distorted by any number of defense mechanisms. Avoidance, denial, repression, suppression, intellectualization, rationalization, projection, splitting, and reaction formation may all contribute to the conscious or unconscious underreporting of symptoms attributable to unresolved abortion issues.

Active defense mechanisms are also the most likely explanation for selection bias and the high rate of concealing abortion history found in national longitudinal studies. Typically, respondents will report under half, and as few as 30%, of the number of abortions expected compared to age-adjusted national data on abortion rates.^106,185,186^

In case series studies, where women are first contacted while at the abortion provider and asked to participate in a follow-up evaluation, both the initial refusal and subsequent dropouts usually exceed 50%.^39,187^ In the Turnaway study, for example, only 37.5% of women asked to participate agreed, and of those who agreed 15% immediately dropped out *before* the first baseline interview, approximately 8 days after the abortion.^179^ The study continued with phone interviews every 6 months for 5 years. Women were rewarded with a US$50 gift card each time they completed an interview. But despite this motivation, by the end of the 3 years, only 27% of the eligible women were participating, and this dropped to only 18% at the 5-year assessment.^188^ Given this high rate of self-censure, the researchers’ conclusion that “Women experienced decreasing emotional intensity over time, and the overwhelming majority of women felt that termination was the right decision for them over three years”^179^ clearly overstates what the Turnaway data can actually reveal. Unfortunately, the authors’ overgeneralized conclusion inspired many newspaper headlines which definitively proclaimed that the overwhelming majority of women are glad they had their abortions.^178,189^ But if the researchers’ conclusions had been more accurately narrowed to describe their actual pool of respondents, the abstract should have read, “Of the 27% of eligible women participating at a three year assessment, the overwhelming majority felt that termination was the right decision for them.” That single clarification would have helped even the most pro-choice reporter to recognize that the views of a self-selected minority of volunteers (27%) simply cannot tell us what the “majority of women” feel and think. What “most women” experience is simply unknown when the majority of women are refusing to share their thoughts and feelings at any given time.

Avoidance, and other defense mechanisms, clearly works. Research has shown that the subset of women who anticipate the most difficulty dealing well with their abortions are right; they do have higher rates of negative reactions.^56^ It is therefore natural for women who anticipate more negative reactions to avoid follow-up surveys that may aggravate those negative feelings. Indeed, one reproductive history survey that included as the last query, “Answering this survey has been emotionally difficult or disturbing,” found that women admitting a history of abortion were significantly more likely to feel disturbed by participating in the survey.^183^ This finding is especially important relative to research designs that rely on waves of multiple interviews over time. Clearly, women who feel more stress at one wave may be more likely to decline to participate again in subsequent waves.

These findings are consistent with studies showing that women refusing to participate in follow-up studies are likely at greater risk of negative reactions to their abortions.^174,190^ While one study has asserted that the women dropping out are not significantly different than subjects retained,^39^ this conclusion was based on demographic comparisons, not on comparison of the presence of risk factors that are more predictive of negative reactions. The authors’ refusal to allow reanalysis of their data^140^ also diminishes the reliability of their conclusions.

Notably, the act of avoiding a post-abortion evaluation may itself be evidence of a post-traumatic stress response. A study of 246 employees exposed to an industrial explosion revealed that those employees who were most resistant to a psychological checkup following the explosion had the highest rates and most severe cases of PTSD. Without repetitive outreach and the leverage of an employer mandate for undergoing post-traumatic assessments, 42% of the PTSD cases would not have been identified, including 64% of the most severe PTSD cases.^191^ In the subsequent clinical treatment of these subjects, the author noted that “In the clinical analysis of the psychological resistance [to the initial assessment] among the 26 subjects with high PTSS-30 scores, their resistance was mainly found to reflect avoidance behavior, withdrawal, and social isolation.”^191^

Our understanding of defense mechanisms also suggests there may be cases where the denial of a link between abortion and abortion-specific symptoms is evidence of both avoidant behavior and an elevated risk of mental illness. It seems likely that defense mechanisms may contribute to a significant underreporting of negative reactions, especially in survey responses. Conversely, questionnaire-based reports may also lead to the exaggerated rating of some positive reactions due to splitting or reaction formation. In these cases, women trying to focus on the positive may respond in ways that may anticipate, or even inflate, the positive feelings *they want to feel* while “rounding down” negative reactions which they want to escape or deny.

The statistical impact of defense mechanisms is also double edged. First, self-censure, dropouts, and concealment of past abortions are all likely to suppress measurements of the prevalence rate of mental illnesses among those volunteers admitting to a past abortion. Second, comparison groups that include women who conceal their history of abortion (who are most likely to have AMH effects) are likely to have inflated prevalence rates for mental illness due to the misclassification of women with a history of abortion into the comparison group of women who, according to the study design, have not been exposed to abortion.^184^ Both problems suggest that odd ratios and prevalence rates based on studies relying on voluntary self-reporting of abortions will most likely be skewed toward underestimating the true risks associated with abortion.

It is also worth noting that defense mechanisms may also impede the ability of women to receive good follow-up care. In a survey of women reporting that they sought post-abortion counseling from a psychologist, psychiatrist, social worker, or other professional counselor, 58% reported that the counseling was not helpful.^45^ Many reported that their therapists simply refused to seriously consider abortions as significant. This phenomenon may be at least partially due to defense mechanisms employed by healthcare professional professionals themselves. Many therapists may have unresolved issues with their own history with abortions; others may be loath to reconsider the wisdom of their advice to previous patients, reassuring them that abortion was a good; still others may have ideological commitments to abortion rights which conflict with their ability to trust their patient’s self-assessments, and some may simply have an uncritical confidence in the widely spread, but exaggerated claim, that “there is no evidence that abortion has any mental health risks.” This is yet another reason why better research and training regarding how abortion may contribute to problems for “*at least some women*” is important to prepare healthcare workers to be more sensitive and open to providing informed care.^45^

### There is no perfect control group; yet all comparison groups provide insights

Since it is impossible to randomly assign women to different groups to be exposed to abortion or not, there are no true control groups in relation to abortion among humans. Given this limitation, comparisons to other groups of women who have not been exposed to abortion are the only option. While no comparison group is perfect,^192–194^ nearly every comparison can be useful for teasing out patterns that may help to inform patients and caregivers regarding the many varieties of abortion experiences.

Comparisons have been made to each of the following: the general population of women,^77,195^ women who have never been pregnant,^94^ women with no reported history of abortion,^74,84,85,91,92,94,95,100,101^ women giving birth,^30,69,71–73,75–77,81,83,86–90,94,97–99,102^ women giving birth to a first pregnancy,^69,86,113^ women having miscarriages or other involuntary losses,^81,88,91,94,195–197^ women experiencing both births and pregnancy loss (abortions or miscarriages),^69,82,107^ women giving birth to unintended pregnancies,^69,72,75,76,86,90,92,98^ and women denied abortions.^179,198^ Together, these findings show that women with a history of abortion are statistically more likely to experience significantly more mental health issues relative to every comparison group that has been examined.

Notably, most of these comparisons are based on general-purpose longitudinal cohort studies. As discussed previously, due to the temporal limits, cross-sectional data, self-selection bias, concealment, and the misclassification of women with an abortion history into the comparison groups, the results of these studies most certainly skew toward underestimating the true relative risks between the groups compared. Still, while every choice for a comparison group is imperfect,^192,193^ below we will argue that there are valid insights that can be gained by every comparison. Acting on that premise, many researchers have chosen to simultaneously compare women who abort to multiple other groups whenever the data allow it.^72,88,92,94^

By contrast, Charles et al.,^6^ have argued that the only “appropriate” comparison group for AMH studies is to women who have “unwanted deliveries.” But this argument is weak for three major reasons.

First, the efforts to define and evaluate what constitutes an “unintended” or “unwanted” pregnancy are themselves imprecise, rendering any study based on such a flawed definition imprecise.^15,199^ Moreover, not intending to become pregnant at a particular time in one’s life is very different than not wanting a child. Indeed, over half of unintended pregnancies are carried to term, accounting for approximately 37% of all births.^200^ Conversely, among women having abortions, the evidence suggests that between 30% and 63% of aborted pregnancies were intended, wanted, welcomed, or involved significant emotional attachment.^48,50,51,148,172^ In short, both groups (women having abortions and women carrying unintended pregnancies to term) encompass a huge variation in intentionality, wantedness, and attachment to their pregnancies.

Second, as Romans^192^ has convincingly argued, the differences in women who choose to carry an unintended pregnancy to term and those who abort are simply immeasurable. No conceivable comparison between the two groups can control for all the possible variations between them. Still, as both the TFMHA^4^ and Fergusson et al.^193^ have argued, even imperfect comparisons have and can continue to yield valuable insights regarding the differences between the women who cope well and those who cope poorly. While such findings cannot tell us what “most women” experience, they can tell us how different subgroups of women compare to each other. These findings are meaningful and actionable since they should be used to guide pre-abortion screening and counseling and post-abortion care^25^ and for informed consent procedures.^23^

Third, the argument for discounting studies that lack information on pregnancy intention appears to have been advanced primarily as an excuse to denigrate the majority of studies on AMH. This charge is supported by the fact the “quality scale” created by Charles et al.^6^ required deducting two of the five possible quality points from any study using any control group other than women carrying unwanted pregnancies to term.

The highly biased and subjective application of Charles et al.’s quality scale is demonstrated by the fact that they rated studies published by AMH minimalists^69,92,201^ using exactly the same national longitudinal data sets as AMH proponents^72,86,101^ consistently higher in quality. Moreover, Charles et al.’s quality scale totally ignored the problem of high concealment, misclassification, and drop-out rates in the very same studies they rated as better. Thus, by ignoring issues related to selection bias, the Charles et al. contrived ranking scale identified just four studies as “very good”—even though three of these had concealment rates of 60% or higher,^185^ and the fourth had a dropout rate of 65%.^76^ Meanwhile, their skewed scale allowed them to rank as “poor” or “very poor” literally all record linkage studies, which by their nature have *no concealment or selection bias*,^81,87,89,97,196^ even though these same studies revealed some of the strongest associations between AMH problems.

The fact that Charles et al.’s study quality scale was deliberately skewed to serve the AMH minimalists’ perspective is perhaps best demonstrated by the fact that when the very same record linkage studies rated as poor by Charles et al. are rated using the Newcastle-Ottawa Quality Assessment Scale (NOQAS) for cohort studies,^202^ a standard and widely used assessment tool across all disciplines, all receive very high scores, 8 or 9, on the NOQAS 9-point scale for quality.^203^

In response to Charles et al.’s argument that the only appropriate comparison group is to women carrying unintended pregnancies to term, the following arguments are made in defense of other comparison groups. I argue that, while no comparison is perfect, every option for a comparison group can be a useful tool in developing a multidimensional perspective on the complexity of AMH issues.

**Figure 3.**
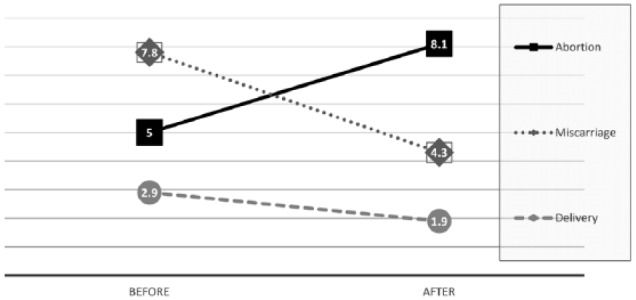
Suicide attempt rates per 100,000 women before and after designated pregnancy outcome. Source: Morgan et al.^81^

First, comparisons to women with a history of abortion and the general population of women provide a useful baseline, especially when combined with comparisons to women who miscarry or carry to term. For example, a record linkage in Finland revealed that the age-adjusted risk of death within a year of pregnancy outcome was 5.5 per 100,000 deliveries, 16.5 per 100,000 miscarriages, and 33.8 per 100,000 abortions, compared to 11.8 per 100,000 age-adjusted women years for the general population of women not pregnant in the prior year.^196^ A similar record linkage study of the population of Denmark revealed a dose effect, with the risk of death increasing by 45%, 114%, and 191% with exposure to one, two, or three abortions, respectively.^112^ Yet another record linkage study examining attempted suicide rates before and after pregnancies revealed declining rates of suicide attempts after both delivery and miscarriage, but a sharp increase in attempted suicide following abortion, as seen in Figure 3.^81^

Comparisons to women who have never been pregnant (nulligravida) are especially important when the aborting women have no live born children.^74,92,94,113,204^ Indeed, this is an important comparison since an abortion of a first pregnancy is essentially an effort to return a woman to her never been pregnant state. Differences between childless women with a history of one or more abortions and those without any history of pregnancy may provide valuable insights into the effects of an interrupted pregnancy on women’s emotional and physical health.

Another important comparison is between women who have induced abortions and women who miscarry. Both have experienced the effects of pregnancy, which may produce long-lasting changes to the brain,^150,205,206^ and maternal attachment.^151,152,154,207^ While the physiological processes of natural miscarriage and induced abortion are different, there may be similarities in the recovery process. Moreover, this comparison may allow insights into the psychological differences between intentionally choosing the end of a pregnancy versus an unintended loss, both of which may be experienced as a form of disenfranchised grief.^45,161^ Arguably, examining the differences between miscarriage and abortion may be the most relevant and important comparison.^203^

Comparisons to women giving birth are also meaningful. Just as a comparison to a never pregnant woman attempts to estimate how closely induced abortion achieves the goal of “turning back the clock” to the point before the woman became pregnant, a comparison to a delivering woman seeks to estimate how a woman’s mental health would fare if she chooses to “move into” the group of women giving birth.

Comparisons between women aborting a first pregnancy and women carrying a first pregnancy to its natural conclusion (birth, miscarriage, or neo-natal loss) are extremely valuable. By excluding the confounding effects of multiple pregnancy outcomes, these studies offer at least a small window on the effects associated with exposure to a single pregnancy outcome. Moreover, they are the proper starting point for investigating the interactions between multiple pregnancy outcomes. This is important since significantly different outcome patterns have been observed relative to multiple pregnancy outcomes and their sequences, including both multiple losses and losses followed or preceded by live births.^88,94^

While comparisons of first pregnancy outcomes are valuable, it should be noted that it is a very poor methodological choice to include in the group of women experiencing a “first live birth” women who are known to have had one or more abortions before their first live birth or between the birth and the date of the mental health assessment.^69,107^ Unfortunately, these flawed studies^69,82,107,208–210^ ignore the extensive evidence showing that a history of pregnancy loss (abortion or miscarriage) is associated with higher rates of mental health problems during subsequent pregnancies.^78,80,99,100,170,211–226^ By adulterating the “control” group of women having a “first live birth” with women who also have a history of one or more abortion and/or miscarriages, the resulting analyses clearly confound rather than clarify the differences between abortion, miscarriage, and childbirth, shifting the known negative effects associated with prior pregnancy losses into results associated with a first childbirth.^69,82,107,208–210^ Arguably, this confounding methodology has been specifically employed by AMH minimalists precisely with the intent of producing results that obfuscate the mental health effects associated with abortion while inflating the effects associated with childbirth.^141,227^

As will be discussed further, we recommend that the best practice for all studies examining the interactions between mental and reproductive health is to include stratification of results by the order and number of exposures to births, abortions, miscarriages, and other pregnancy losses.^94,141,227^ Otherwise, the effects of different pregnancy outcomes are likely to be obscured rather than clarified.

In addition, we would note that the argument of Charles et al. for discounting studies that lack controls for pregnancy intention may do a major disservice to both women considering abortion and their caregivers. For all the reasons given above, the best evidence indicates that reasonable patients may consider any and all of the comparisons discussed above to be of value in their efforts to evaluate the potential risks and benefits of an abortion in their own personal circumstance^23,25^

Finally, it has been argued that the differences between women who abort and those who do not are so extreme that the only meaningful comparison is between women who abort and women who sought but were denied an abortion.^194^ While this comparison might be informative, it is clearly not a perfect comparison since the reasons why women may end up being denied an abortion are also likely to make these women significantly different than the average woman seeking and obtaining an abortion. Moreover, since in most countries where abortion is legal, very few women are denied an abortion undertaking such studies may be impractical. Indeed, the only set data set using this control group is the so-called Turnaway Study. Indeed, the argument that this is the only valid comparison group appears to be made in an attempt to dismiss all other research in favor of this single data set. But there are many problems with the Turnaway Study data set.^198^ The most damning is the problem of self-censure. Over 70% of women approached to participate in this study refused, even after they were promised payments for participating, plus, nearly half of those who did participate subsequently dropped out.^198^ This high refusal rate alone renders the Turn-Away Study data meaningless in terms of drawing any conclusions regarding the general population of women seeking or having abortions, and that is just one of many major flaws in the Turnaway Study methodology and execution.^198^

### Poorly defined terms produce misleading conclusions: unwanted, relief, and more

Unfortunately, a great deal of the literature on AMH revolves around poorly defined terms. The resulting lack of precision and nuance contribute to AMH minimalists and AMH proponents talking past each other and contributes to overgeneralizations regarding research findings, especially in the press releases and position papers of pro-choice and anti-abortion activists.

As previously discussed, one common overgeneralization is the assertion that abortions typically involve “unwanted” pregnancies. A closer look, however, reveals that many aborted pregnancies, perhaps the majority, occur for planned, partially wanted, or initially welcomed pregnancies.^48,50,51,148,172^ By “welcomed” pregnancies, I mean pregnancies which were not planned in advance but to which the woman was open or naturally inclined to accept and embrace if only she had received the support of her partner, family, or others.^45,181,228^

Attempts to define “unwanted” pregnancies are also complicated by the fact that many women report a divide between their emotional and intellectual responses when they first discover they are pregnant. Emotionally, they may be excited that a new life is growing inside them and may fantasize about having the child. But at the same time, their logical side may be immediately convinced that abortion is their only pragmatic choice.^45^ The pregnancy may therefore be simultaneously “emotionally wanted” and “logically unwanted.”

Based on both clinical experience and case series studies,^173^ we hypothesize that many delayed reactions to abortion stem from the psychological conflicts that arise when emotions are suppressed in favor of pragmatic choices. In such cases, forward-looking women with strong defense mechanisms are likely to cope well with their choice for many years. But if this coping is achieved by suppressed emotions, this may consume energy and may even fuel maladaptive behaviors, like substance use and sleep disorders. Any connection between these symptoms and underlying abortion associated conflicts may not be recognized until some subsequent event or stress compels a reexamination of unresolved maternal attachments or the woman’s moral priorities.

One measure of openness to having a child, seldom addressed in AMH studies, is desire for children at some later date. A high level of desire for future children suggests that an aborted pregnancy was most likely problematic due to specific circumstance or lack of sufficient social support. Among a sample of women seeking counseling for post-abortion distress, 64% felt “forced by outside circumstance” to have an abortion and 83% indicated they would have carried to term if significant others in their lives had encouraged delivery.^181^ While statistics gathered from women contacting post-abortion recovery programs may be not representative of the general population of women, these findings demonstrate that labeling these aborted pregnancies as “unwanted” does not reflect the experience of the women who subsequently do seek post-abortion help.

Given the wide variation in levels of intention or openness to pregnancy, much more extensive data on intention^199,228^ and attachment^207^ are required to draw any conclusions regarding the mental health effects of abortion relative to *various levels* of women’s attachment, intention, and outcome preferences.

A second poorly defined variable is “relief.” AMH minimalists have frequently asserted that the most common reaction to abortion is relief.^4^ But “relief” is a very broad term. A woman reporting “relief” may be referring to (a) relief that she will not have a baby, (b) relief that a dreaded medical procedure is now behind her, (c) relief that her parents will not discover she was pregnant, (d) relief that her partner will finally stop harassing her to have an abortion, or (e) any number of other reasons for feeling a reduction in stress.

But as indicated earlier, abortion can be both a stress reliever and a stress creator. The many declarations by AMH minimalists that “relief” is the most common reaction to abortion tend to distract the public from the fact that the vast majority of women reporting relief are also reporting a host of negative feelings at the same time.^39,50,62^

Similarly, claims that “the most common reaction” to abortion is relief is also misleading because it falsely suggests that a truly representative sample of all women having abortions have been queried about their most prominent and common reactions. But in fact, all the case series studies assessing “relief” have self-censure and dropout rates exceeding 50%.^39,59^ When only a minority of women agree to report on their reactions to an abortion, these studies cannot reliably tell us anything about the majority of women. This is especially true if the self-selection bias is toward women who expect to feel more relief because their abortion decision is more consistent with their own desires and preferences, while those who refuse to participate anticipate and do experience more negative reactions.^174,190,191^

Another misleading factor is that relief is most often reported as a single variable whereas negative reactions are often averaged together. For example, one of the most frequently cited case-series reporting that women felt “more relief than either positive or negative emotions” was based on comparing the results of a single question regarding relief to an average of six scores (“sad,” “disappointed,” “guilty,” “blue,” “low,” and “feelings of loss”) chosen to represent negative emotions and an average of three scores (“happy,” “pleased” and “satisfied”) chosen to represent positive emotions (excluding relief).^39^ This methodology was highly problematic.

While it would be interesting to see score distributions for each reaction separately,^45^ how can a variety of emotions be “averaged” together in any meaningful way? For example, if a score of 1 (corresponding to “not at all” on the Likert-type scale used) is equivalent to 0% of the relevant emotion and a 5 (“a great deal”) is 100% of that emotion, averaging six emotion scores together presumes that a rating of 3 (50%) for “disappointed” is truly equivalent to twice a rating of 2 (25%) for “feelings of loss” and half the value of a rating of 5 for “guilty.”

But what makes this averaging process even more suspect is that the least common negative reaction (“disappointed,” perhaps) would dilute the entire average of negative reactions, concealing the frequency of the more common reactions (“guilty,” perhaps). Most importantly, while the most common negative and positive reactions were diluted by this “averaging” process, the “relief” score was not subject to the dilution by averaging with any of the other positive emotions.

Yet another problem with the authors’ conclusion^39^ was their presumption that the six negative reactions they asked about are actually the most common negative reactions. But three of the six negative reactions (“sad,” “blue,” “low”) appear nearly synonymous. The similarity of these three may have been deliberate in order to boost the reliability score for the authors’ scale. One of the remaining choices, “disappointed,” is simply odd, rather bland, and perhaps disinviting as it is not a term that has been reported in interviews with women reporting negative reactions to abortion. ^45,172,173,181^ While the assessments of “guilty” and “feelings of loss” were appropriate, it would have been more illuminating to report these separately rather than in an “average” of negative emotions.

In any event, averaging emotion scores is problematic and in this case the choice of the six negative feelings chosen to be averaged together failed to include many of the negative emotions most commonly reported in surveys of the women who seek post-abortion counseling, including sorrow, shame, remorse, emptiness, anger, loneliness, confusion, feigned happiness, loss of confidence, and despair.^45^

Despite the many limitations regarding the claim that “relief” is more common than negative reactions, it is notable that the same researchers also found that between the 3-month and 2-year post-abortion assessments, both relief scores and positive emotions decreased significantly while the average for negative emotions increased.^39^ In other words, even with a self-selected sample of women most likely to have more positive reactions, those positive emotions declined and negative emotions increased within the first 2 years. If that trend continued over 20 years, the finding that the “most common reaction” to abortion was relief may not have held up over a longer period of time.

Similar problems apply to the widely reported claim that most women are satisfied with their decisions to abort.^179^ In this case, the self-selection bias was profound, with only 27% of the eligible women participating at the date of their first assessment. In addition, this “finding” was based on a binary yes or no response to a single question: “Given your situation, was your decision to have an abortion right for you?” This question clearly invited reaction formation and splitting. Additional questions, such as, “If you had received support from others, would you have preferred to have carried to term?” would have provided deeper insight into the participants’ true preferences.

Despite the problems with their methodology and self-selected sample, these researchers’ confident assertion that the vast majority of women are satisfied with their abortions generated bold headlines.^189^ But these misleading headlines were clearly based on poor science.^198^ Similar questions, posed to a different self-selected sample of women seeking post-abortion counseling, reveal that 98% of that sample of women regret their abortions.^45^ These resuts are contradictoruy because neither of the two samples just cited represent the general population of women having abortions. Given the fact that so many women refuse to respond to questionnaires about their abortions, it is impossible to ever be certain what “the majority” of women feel or think about their past abortions at any given time, much less through their entire lifetimes.

If there is any consistency in the evidence, it is in regard to the finding that satisfaction declines and regrets increase over time.^38,39,45^ Therefore, the existing data for claims regarding high levels of relief and decision satisfaction are highly questionable in the short term and meaningless in regard to predicting feelings in the long term.

Is abortion the sole cause, a contributing cause, or never a cause of mental health problems? Or is this question just a distraction from helping women?

Normally, the burden of proving that any proposed medical treatment produces real benefits which outweigh any risks associated with the procedure falls on the proponents of the treatment.^229^ Indeed, proponents of a treatment are also tasked with the obligation of proving not only specific benefits but also with identifying the symptoms and circumstances for which the treatment has been proven to be beneficial and those cases for which it might be contraindicated. After all, no treatment is a panacea. Even highly successful elective treatments such as Lasik are contraindicated for 20%–30% of patients considering the surgery.^230^

Evidence-based medicine is centered on the idea that there must be real evidence of benefits that outweigh the risks associated with a medical intervention. But there are no statistically validated medical studies showing that women facing any specific disease or fetal anomaly fare better if they have an abortion compared to similar women who allow the pregnancy to continue to a natural outcome.^17,231,232^ Nor is there evidence of any mental health benefits.^17,25^ As a result, in approaching a risk–benefits assessment, there are literally no studies to place in the benefits column of an evidence-based risk–benefits analysis. Conversely, there are literally hundreds of studies with statistically significant risks (both physical and mental) associated with abortion which must be considered in weighing abortion’s potential risks against the patient’s hoped for benefits.^11,112,113,232,233^ See, for example, the references to Table 1.

In this regard, induced abortion is an anomaly. It is the only medical treatment for which the principles of evidence-based medicine are routinely ignored, not for medical reasons, but by appeals to abortion being a fundamental civil right^135^ or a public policy tool for population control.^25^ From these vantage points, there has arisen an *a priori* premise that abortion should presumed to be safe and beneficial. Therefore, according to defenders of abortion, the burden of proving the safety and efficacy of abortion is no longer on them. Instead, abortion skeptics must prove that abortion is the sole and direct cause of harm to women—and not just a few unfortunate women, but a large proportion of women.^4,6,57^

This difference in evaluating abortion compared to other medical treatments was at the center of a Planned Parenthood suit challenging a South Dakota statute requiring abortion providers to inform women of research regarding psychological risks associated with abortion. Abortion providers argued that there was not yet enough proof that abortion was the “direct cause” of the statistically significant higher risks of mental illness, including suicide, following abortion. Therefore, they argued, disclosing the findings of these studies to women might unnecessarily frighten their patients.^234^ But the Eighth Circuit United States Federal Court of Appeals rejected Planned Parenthood’s argument, ruling that it was a standard practice in medicine to “recognize a strongly correlated adverse outcome as a ‘risk’, even while further studies are being conducted to investigate which factors play causal roles.”^234^ The court went on to add that Planned Parenthood’s “contravention of that standard practice” had no legal merit since “there is no constitutional requirement to invert the traditional understanding of ‘risk’ by requiring, where abortion is involved, that conclusive understanding of causation be obtained first.”^234^

This appellate court’s ruling is consistent with idea that “risk,” by definition, includes uncertainty—otherwise, it would not be a “risk” but rather a “certainty.” Therefore, the question of whether a statistically significant risk is *solely due* to abortion, *partially due* to abortion, or *only incidentally associated* with abortion is itself just another of the uncertainties about the procedure, and therefore a true risk about which patients should be informed.^25^

The court’s decision favoring disclosure of all risks, even when causality is challenged by proponents of the procedure, is in line with the preferences reported by 95% of women considering elective medical procedures, to be informed of “all possible complications.”^23^ From a feminist perspective, the right of each individual woman to evaluate for herself whether a statistically significant risk is incidental or causal would also appear be central to the protection of each woman’s personal liberty. Indeed, the United Nation’s Fourth World Conference on Women’s Declaration and Platform for Action, which specifically addressed the issue of unsafe abortions, urged every government to

Take all appropriate measures to eliminate harmful, medically unnecessary or coercive medical interventions, as well as inappropriate medication and over-medication of women, and ensure that all women are fully informed of their options, including likely benefits and potential side-effects, by properly trained personnel.^235^ (Emphasis added)

For the reasons above, the claim that the higher incidence rates of mental health problems associated with abortion are most likely “spurious”^105^ has no bearing on informed consent. Only after full disclosure can each patient judge the relevance of such information for herself.

These challenges are also irrelevant to the obligation of the treating clinician to screen for the risk factors associated with higher rates of negative outcomes associated with abortion.^23,25^ After all, even if abortion proponents could prove that 100% of all the negative effects associated with abortion are causally due to common risk factors, the finding that abortion is consistently associated with higher rates of mental health problems^15,57,82,89,94^ is still an actionable *marker* that can and should be used to identify women who may benefit from referrals for additional counseling.^26,27,30,32–34,36–39^

Still, the question of causation is worthy of additional attention. One approach for judging causality is to apply the nine criteria Bradford-Hill proposed to identify the causal role that occupational and lifestyle factors may play in the development of diseases, such as cancer. These include temporal sequence, strength of association, consistency, specificity, biological gradient (dose–effect), biologic rationale, coherence, experimental evidence, and analogous evidence.^236^ Applying the Bradford-Hill criteria to the AMH question, Fergusson, a pro-choice proponent, concluded that “the weight of the evidence favors the view that abortion has a small causal effect on the mental health problem.”^75^

It should be noted, however, that the Bradford-Hill criteria were developed to evaluate contributing factors for physiological diseases. Bradford-Hill therefore ignored a type of evidence for causality which is unique to psychological diseases, namely, self-aware attribution of causal pathways. For example, the evidence of a woman who says, “After the death of my child, I drank more heavily to dull the pain,” is a conscious identification of cause and effect regarding her own mental state and behaviors.

Indeed, in the psychological sciences, it has been a traditional practice to begin any investigation of mental illness by first listening to those individuals who claim they have a psychological problem. After carefully listening to a “sick” population, psychologists can then map the range of reported symptoms and then build hypothesis regarding the contributing factors and causal pathways which can then be explored by surveys of the general population. This was the approach AMH proponents used in their initial investigations of women seeking post-abortion counseling.^45,171,181^ Because these samples were based on women experiencing post-abortion issues, they were likely skewed toward the Allie All-Risks archetype. Still, because they were focused on developing a profile of the women having post-abortion issues, this was a valid starting point for identifying the most common complaints and recurring patterns.

By contrast, most AMH minimalists have tested their hypotheses using surveys of women contacted at abortion clinics. These survey instruments appear to have been developed with little or no attention to the complaints of the women who reported post-abortion mental health crises. Moreover, because these surveys are implemented in cooperation with abortion providers, in a stressful situation during which less than half of the women agree to participate, it is likely that these self-selected samples skew toward the Betsy Best-Case archetype.^39,237^

Even though AMH minimalists and proponents approach their research from different perspectives, the results from both sides consistently show that at least a minority of women experience mental health problems that they attribute, at least in part, to their abortions. While not included in the Bradford-Hill criteria, when it comes to mental health issues, *the fact that so many intelligent, self-aware women attribute specific patterns of emotional distress to their history of abortion* is one of the strongest pieces of evidence that abortion directly contributes to mental health problems. The same is true with regard to mental health associated with miscarriage. The validity of this evidence is further strengthened by the professional assessment of both pro-choice therapists^40–44^ and pro-life therapists^45–47^ who also attest to the causal connection.

Similarly, the clinical evidence that women struggling with post-abortion mental health issues improve following treatment focused on their abortion loss^40,46,238–240^ also supports the conclusion that abortion can cause, trigger, or exacerbate psychological illness. After all, a successful treatment is evidence in favor of a correct diagnosis.

As previously noted, self-attribution is not perfect evidence. Defense mechanisms often operate by obscuring the “true cause” of one’s mental distress. But we would argue that the bias of defense mechanisms would be toward underreporting of effects truly associated with an abortion rather than toward false attribution of unrelated effects to past abortions.

That is not to say that pre-existing mental health issues cannot become intermingled with an abortion. To the contrary, clinical experience shows that abortion can become such a significant stressor in a woman’s life that other pre-existing issues can become enmeshed in the abortion and its aftermath. Pre-existing substance abuse, for example, may become intensified in the abortion aftermath, but it would be a self-deception to blame the abortion entirely for such substance abuse. On the contrary, once the issues become intermeshed, progress in dealing with underlying issues will be hindered by a failure to address the intermingled abortion issues.

Similarly, even in cases where suicide notes specifically attribute a woman’s final act of despair to her recent abortion,^241^ other pre-existing factors may also contribute to these tragedies. In short, while it would be absurd and insulting to deny that abortion at least *contributes* to such suicides, it would be a mistake to assume that abortion is the sole cause of suicide or any other specific mental illness.

As stated previously, abortion does not occur in isolation from interrelated personal, familial, and social conditions that influence the experience and mental health of each individual. Moreover, there are likely a multiplicity of different pathways for effects to manifest either in the near or longer term.^18^ In general then, abortion is most likely a *contributing factor* to the manifestation of problems rather than *the sole factor*. It may be trigger latent issues, intensify or complicate existing issues, interact with pre-existing issues to create new issues, or contribute in any number of ways unique to any particular individual’s susceptibilities and prior and subsequent life stresses.

In summary, there is incontrovertible evidence that abortion contributes to mental health problems, both directly and indirectly. Based on reports of clinical experience, it would appear that abortion can be the primary cause for mental health issues in some women. But it may also trigger, intensify, prolong, or complicate pre-existing mental health issues. Still, for the sake of argument, assuming AMH minimalists are right in their assumption that abortion itself is never the “sole cause” of mental health problems, there is still no reasonable doubt that abortion *contributes to mental health issues* in some women.

Finally, it should be emphasized that the difficulties involved in proving causality cut both ways. The burden of proving the efficacy and safety of abortion falls on abortion providers. To date, they have failed to provide any evidence, much less proof, that abortion is the *sole and direct cause* of any health benefits for women in general, or even for specific subgroups of women.^193,232^ Nor have they shown that the benefits women hope to obtain through abortion are proportionate to or greater than the significantly elevated rates of negative outcomes associated with abortion. In this regard, abortion continues to be an experimental treatment, one for which they hoped for benefits are unproven. And with no proven benefits, the risks–benefits ratio is unknown even for those women without any known risk factors.

### Is it reasonable to attribute all negative effects to pre-existing factors?

There is no longer any dispute regarding the fact that, on average, women with a history of abortion have higher rates of mental illness compared to similar women without a history of abortion. But AMH minimalists frame this admission in the context of arguing that this is most likely due to pre-existing mental health issues.^5,6,242^ In other words, they argue that a higher percentage of aborting women were “already emotionally broken” to begin with. Therefore, higher rates of mental illness following abortion are just a continuation of pre-existing mental frailty.

This argument is indistinguishable from the centuries-old accusation of personal defects applied to “hysterics,” “malingerers,” “cowards” and others who exhibit traumatic reactions.^45,243^ This blame-their-weakness argument is just a corollary to the assertion that higher quality, more emotionally stable people simply do not break under such circumstances.

In courtrooms, this line of arguments is known as the thin skull, or eggshell skull, defense. It asserts that a defendant should not be held accountable for injuries that would not have been suffered if the plaintiff had not been predisposed to injury due to pre-existing physical or emotional defects. Notably, the thin skull defense has been rejected in most legal jurisdictions. Even if the damages of the “frail” plaintiff are greater than they would be for a healthier person, jurists have ruled, the defendant is still liable for the greater damages because

a defendant who negligently inflicts injury on another takes the injured party *as he finds her*, which means it is not a defense that some other person of greater strength, constitution, or emotional makeup might have been less injured, or differently injured, or quicker to recover.^244^ (Emphasis added)

Applying the thin skull legal analysis to abortion, this means that a physician who fails to screen for known risk factors, such as prior mental illness, before recommending or performing an abortion is guilty of negligence if the woman suffers any subsequent mental health problems because it is precisely the obligation of the physician to treat the woman “as he finds her.”

In short, the argument that negative effects may be mostly due to pre-existing mental health problems simply strengthens the argument for better pre-abortion screening for this and other risk factors.^12,25,26,32^ Conversely, it does not at all support the presumption that abortion is safe or likely beneficial to most women, much less all.

The “broken women” argument has also been used by AMH minimalists to argue that the emotionally fragile women having abortions would most likely face as many or more mental health problems if they were denied abortion.^245^ But again, this argument is based entirely on conjecture. While only a few studies have examined the mental health of women denied abortions, none have found any significant mental health benefits compared to other groups of women.^76,188^

Still another AMH minimalist argument is that women with prior mental illness may instinctively know they are less likely to cope well with an unwanted pregnancy, so the higher rate of abortion among women with mental illness is actually a sign of these women choosing abortion wisely.^106,107^ Again, this is entirely speculation. It ignores the likelihood that mentally ill women, especially those with a history of being abused, may simply be more susceptible to being pressured into unwanted abortions^45^ like Allie All-Risks. Moreover, it ignores the ethical obligation of caregivers to discourage, rather than enable, patterns of behavior that may be self-destructive.

Rather than just assume that mentally ill women are wisely inspired to choose abortion more often than mentally healthy women, would it not be best to screen women seeking abortions for mental illness so women can be counseled in a manner that more fully addresses their needs in the context of their mental illness?^25,36^ As previously noted, while abortion may relieve some stresses, it may also create new ones.

Moreover, bearing children may actually contribute to mental health improvements through direct biological effect,^150,205,206^ by expanding and strengthening interpersonal relationships with the child(ren) and others,^151,152,154,207^ or by behavioral adaptations that may replace risk-taking with self-improving behaviors. These benefits may also apply to bearing unplanned children. Indeed, given how common unplanned pregnancies are throughout the millennia, it could be argued that female biology has evolved mechanisms in order to adapt and adjust to unexpected pregnancies.

In short, the argument that higher rates of mental illness following abortion are simply due to mentally ill women being wise enough to choose abortion more often is simply not supported by any statistically validated research. Instead, the opposite argument, that giving birth is more likely to produce mental health benefits, is more plausible and better supported by actual data.

It should also be noted that while we are aware of only one record linkage study examining mental health effects for women *without any history of mental health issues*, that study (by AMH minimalists) revealed that a history of abortion was associated with a significantly increased risk (risk ratio (RR) = 1.18; 95% CI = 1.03–1.37) of postpartum depression after a first live birth.^80^

Closely related to the pre-existing mental illness issue is the finding that women with a history of abortion also have higher rates of abuse and violence in their lives. According to this argument, violence^106,110^ or childhood adversities,^106^ not abortion, are the most likely cause of higher rates of mental illness among women with a history of abortion. This hypothesis is contradicted, however, by studies which have shown that there are higher rates of mental illness associated with abortion even after controlling for violence.^94,109^ More importantly, it is a mistake to engage in either/or arguments; a both/and approach is both more likely and more productive. Clearly, a history of abuse contributes to a heightened risk of both pregnancy and abortion, especially abortions to satisfy the demands of others. At the same time, clinical experience reveals that issues related to abuse and abortion can become deeply entangled. Efforts to treat based on an either/or attribution are most likely to be frustrated. Progress is most likely to be made when *both* the abuse *and* abortion experiences are holistically addressed.^45^

While it important to study the interactions between exposure to violence and abortion on mental health, it is also important to consider that there may be two-way interactions. Surveys of women entering into post-abortion counseling reveal high percentages reporting elevated feelings of anger (81%), rage (52%), more easily lost temper (59%), and more violent behavior when angered (47%) following their abortions, which can obviously increase incidence rates of subsequent intimate partner violence.^45^ Moreover, in the same sample, in which 56% reported suicidal feelings and 28% reported attempting suicide (with over half trying more than once), there are case studies of women “pushing the buttons” of a violent partner because they believed they did not “deserve to live.”^45^ This escalation of violence following abortion may help to explain the elevated rate of homicide among women with a history of abortion.^88,232,246^ For these reasons, given the multiple pathways for interactions between abortion and violence, studies that fail to distinguish between violence before and following abortion are methodologically flawed.^110,247^

While prior abuse and mental health problems receive the most blame for why women with a history of abortion have higher rates of mental illness, a few AMH minimalists insist that the blame for mental illness following abortion can always be shifted to other risk factors.^248^ For example, when Steinberg et al.^30^ found that substance abuse rates were significantly associated with abortion even after controlling for dozens of other risk factors, they dismissed their own findings with the assertion that these effects are most likely due to as yet unidentified common risk factors.

In response, AMH proponents argue that (a) the burden of proving safety and effectiveness is on the proponents of a medical treatment and (b) given the weight of the evidence, it is far more logical to accept that abortion is at least a *contributing* factor that may work in concert with any number of other contributing factors.

In addition, denying that abortion directly contributes to mental health problems is illogical given the fact that so many of the risk factors identified by AMH minimalists themselves (see Table 1) are specifically part of the abortion experience. These include feeling pressured to abort by others; negative moral views of abortion; low expectation of coping well after an abortion; ambivalence about the abortion decision; and feelings of attachment or commitment to a pregnancy that is meaningful or wanted.^25,35,249^

In other words, given what we know of the risk factors associated with mental illness after abortion, many of them are directly enmeshed in the abortion experience; they are not fully independent of the pregnancy and abortion experience. Therefore, even to the degree that mental illnesses can be associated with common risk factors for both unintended pregnancy and abortion, such as a history of sexual abuse, the intermeshing of elevated risk for pregnancy, abortion, and mental health issues precludes the conclusion that abortion *does not contribute in any way* to the observed problems. The only support for that argument comes from ideology, not from any statistically validated studies. For example, an incest victim may be at greater risk of a high school pregnancy with the first boyfriend that she imagines will be able to free her from an abusive step-father.^250^ She may also be at greater risk to being pressured into an unwanted abortion. While it would be a mistake to blame the abortion for all of her subsequent mental health problems, even if a subsequent suicide note focuses on the abortion, it is ludicrous to assert that her abortion did not contribute to her problems. Moreover, it is also evident that the failure of healthcare providers to identify the risk factors that made her a poor candidate for abortion missed an opportunity to assist her in using her pregnancy to break a cycle of exploitation and trauma.

Finally, it should be noted that AMH minimalists frequently cite studies showing that women who deliver an unintended pregnancy have more subsequent problems than women who only have intended pregnancies.^248^ From this base of evidence, they argue that since women who deliver unintended pregnancies have more problems, with mental health and otherwise, it follows that access to abortion helps to reduce the problems associated with unintended pregnancies. But this argument falsely presumes that abortion puts women who have unintended pregnancies back into the category of women who have never had an unintended pregnancy, and that all intended pregnancies are carried to term. But there are not just two groups: (a) women with “perfect” reproductive lives and (b) women with a history of unintended pregnancies. There is a third group, (c) women who have had abortions, who may fare worse than either of the other two groups.

While AMH proponents do not dispute that on average women with unintended pregnancies may face more problems than women who have perfect reproductive lives, it appears likely that they still have fewer problems than women who abort. Indeed, as previously discussed, not a single study has found evidence that the mental health of women who deliver an unintended pregnancy is worse than that of women who have abortions.^69,72,75,76,86,90,92,98,188^ To the contrary, the only statistically significant findings indicate that women who abort are likely to have more mental health problems than those who deliver their unintended pregnancies.^17^

### The controversy over abortion related PTSD is more political than scientific

AMH minimalists often reserve the greatest scorn for statements made by AMH proponents that abortion can be a traumatic experience that may contribute to PTSD.^4,251,252^ But this opposition seems to be driven more by a desire to silence abortion skeptics than to honestly report on the connections between abortion and traumatic reactions as revealed in the literature.

First, it is notable that all pregnancy outcomes are associated with some PTSD risk. Both vaginal and cesarean deliveries can be experienced as traumatic with a corresponding risk of PTSD.^225,253–255^ Miscarriage and other natural pregnancy losses are also consistently associated with increased risk of PTSD.^170,222,256–258^ It should therefore come as no surprise that induced abortion is also consistently found to be associated with the onset of PTSD symptoms.^21,39,50,60,170,225,259–269^ Notably, a history of induced abortion is also a risk factor for the onset of PTSD following subsequent pregnancy outcomes,^170,225,260,270^ so the effects of abortion may not always be immediate but may be triggered by subsequent deliveries or natural losses, or even subsequent non-pregnancy-related events.^271^ These findings are consistent with the insight that multiple traumas and related life experiences may contribute to the triggering of PTSD symptoms.

Given the weight of the many statistically validated studies cited above, much less than the reports of clinicians and women who attribute PTSD symptoms to their abortions, it seems evident that the effort of a few AMH minimalists to categorically deny that abortion can contribute to traumatic reactions is driven by ideological considerations, not science. That said, it should also be noted that not all women will experience abortion as traumatic. Moreover, the susceptibility of individuals to experience PTSD symptoms can also vary based on many other pre-existing factors, including biological differences. So the risk of individual women will vary, as it does for every type of psychological reaction. Still, when even the chair of the APA’s TFMHA has reported identifying abortion-specific cases of PTSD in one of her own studies,^39^ the claim that abortion trauma is a “myth” advanced purely for the purposes of anti-abortion propaganda it itself nothing more than pro-abortion propaganda.^252^

The evidence is clear that some women do experience abortion as a trauma. The prevalence rates and pre-existing risk factors may continue to be disputed, but the fact that abortion *contributes* to PTSD symptoms in at least a small number of women is a settled issue.

## Recommendations for research and collaboration

Good research is essential for both healthcare providers and patients. Better information about the risks and benefits associated with abortion should contribute to better screening, better risk–benefit assessments, and better disclosures to patients,^23^ that will help to shape the expectations of patients and those who advise them. Better information will also improve the identification of at risk patients who may benefit from referrals to post-abortion counseling.

As previously discussed, while the ideological divides between AMH minimalists and proponents will continue to shape how each side interprets the data, these differing viewpoints actually provide an opportunity for improving the collection of useful data, analyses of the available data, and more thorough interpretations of research findings. Therefore, healthcare providers and patients would be better served by AMH minimalists and AMH proponents both bringing their various perspectives to bear on research efforts in a more cooperative fashion.

Whenever possible, research teams should include both AMH minimalists and AMH proponents. Such cooperation would improve methodologies by better addressing the differing concerns of each perspective at the time of the study design. Collaboration in the writing of introductions and conclusions to such studies would also be improved by bringing balance to both perspectives and by reducing the tendency to overgeneralize results of specific analyses.

More specific opportunities for collaboration and better research are discussed below.

### Expanding the research goals

A major problem with abortion research and reviews is a failure to address all of the relevant questions which need to be asked, investigated, and answered. For example, the team from the National Collaborating Center for Mental Health (NCCMH) that wrote a review of AMH issues for the Academy of Medical Royal Colleges in 2011 strictly limited their investigation to only three questions: “(1) How prevalent are mental health problems in women who have an induced abortion? (2) What factors are associated with poor mental health outcomes following an induced abortion? (3) Are mental health problems more common in women who have an induced abortion when compared with women who deliver an unwanted pregnancy?”^5^ Most notably, the NCCMH team chose to ignore the question specifically posed for it to investigate in the 2008 Royal College of Psychiatrists position statement on abortion, namely, “whether there is evidence for psychiatric indications *for* abortion”^272^ (emphasis added). Given the lack of any evidence for psychiatric indications for abortion, it seems likely that the NCCMH decided to ignore this question because it echoed previous allegations that UK law was not being followed in regard to limiting abortion to cases where there are therapeutic benefits.^273^

Many additional questions were raised during the consultation process when the NCCMH team invited comments and suggestions from experts. But all of these questions were summarily rejected by the NCCMH team as being “beyond the scope” of their review, even though they acknowledged that many of these other questions were equally important to the three questions they had chosen.^274^ Indeed, a reading of the consultation report, which was effectively the peer review given to the paper, reveals general dissatisfaction with the three questions chosen by the NCCMH team and with many of their choices in methodology and overstatement or understatement in their conclusions. The consultation report anticipated the many criticisms of the final report^19,275^ and revealed that NCCMH team was not very responsive to the issues and concerns raised during this peer review. Arguably, the NCCMH team’s unstated mission was to protect the status quo, and so they limited themselves to questions and methodological choices that would allow them to achieve that predetermined goal.

The following is a list of some key research questions that should be addressed in future studies and reviews. It was developed, in part, by using the NCCMH consultation report as a starting point:^274^

1. How prevalent are mental health problems in women who carry unplanned pregnancies to term compared to women who deliver wanted pregnancies, to women who have no children, and to women who have abortions?
2. Given that women may experience a range of reactions in the near term and over a period of many years, what are the cumulative rates of negative reactions over a long period of time (including a minimum of 30 years) and what are the temporal, cross-sectional prevalence rates relative to various risk factors that may contribute to these temporal differences?
3. Among women who do experience negative emotional reactions (not limited to mental illness) which they attribute to their abortions, what reactions are reported?
4. What treatments are most effective?
5. What statistically validated indicators predict when the mental health risks of continuing a pregnancy are greater than if the pregnancies were aborted?
6. What statistically validated risk factors predict negative outcomes following one abortion, two abortions, and three or more abortions compared to each available comparison group?
7. What factors, if any, are associated with improved mental health following abortion compared to similar women who carry a similarly problematic pregnancy to term?
8. Among women *with* pre-existing mental health issues, what factors predict a likelihood that abortion may contribute to a *reduction* in mental health problems (intensity, duration, and number of mental health issues), and what factors predict a likelihood that abortion may contribute to an *increase* in mental health problems?
9. Among women *without* pre-existing mental health issues, what factors predict a likelihood that abortion may protect good mental health, and what factors predict a likelihood that abortion may contribute to subsequent mental health problems?
10. Is presenting for an abortion, or a history of abortion, a meaningful diagnostic marker for higher rates of mental illness and related problems that can be timely addressed by appropriate offers of care?
11. In evaluating the risk–benefits profile of a specific patient, what criteria should be met in order to reach an evidence-based conclusion that the benefits of abortion are most likely to exceed the risks?
12. In cases of pregnancy following rape or incest, what are the short- and long-term mental health effects associated with each of the following outcomes: (a) abortion, (b) miscarriage or stillbirth, (c) childbirth and adoption, and (d) childbirth and raising the child?
13. Is abortion associated with an increase in rapid repeat pregnancies, that is, “replacement pregnancies?” If so, what portion are delivered, aborted, or miscarried?
14. Does a history of abortion contribute to the strengthening or weakening of the woman’s relationships with her partner and/or others?
15. What are the mental health effects of the abortion experience, if any, on men?
16. What are the mental health and developmental effects of the abortion experience, if any, on previously born children and/or subsequently born children?
17. Does a history of abortion contribute to or hinder bonding and parenting of previous and/or subsequently born children?

### National prospective longitudinal studies specific to reproductive and mental health

While a number of analyses have been published based on longitudinal studies, none of these studies were designed to specifically investigate the intersection between AMH issues. The need for better longitudinal studies to investigate AMH has been recognized in other major reviews,^4,24,274^ yet the call for such research has not yet been heeded.

We recommend that the value of such longitudinal studies would be vastly increased by expanding the goal of data collection to encompass not just mental health effects associated with abortion but also with all reproductive health issues from first menses to menopause. This would assist in research related to infertility, miscarriage, assisted reproductive technologies, postpartum reactions, premenstrual syndrome, and more. And given the interactions with multiple pregnancy outcomes already seen in AMH research,^88,94,170,203^ comprehensive reproductive health histories are needed in any case.

Most importantly, the design and management of such studies should include both AMH minimalists and AMH proponents. An explicit objective should be ensuring that every line of questioning either side considers important is included. When both sides contribute to the design of such studies and have equal access to the same data, concerns about suppressed findings or incomplete analyses will be dramatically reduced … at least after re-analyses. When both sides have equal access to better data, it is more likely that the areas of consensus will increase.

The value of longitudinal studies would also be enhanced by seeking the consent of participants to link their medical records to their questionnaires. This would be most helpful given the fact that many women are reluctant to reveal abortion information even in responding to a confidential questionnaire. Since women’s willingness to share data may vary over time, this request for record linkage should perhaps be offered multiple times over the course of the longitudinal study. While many will likely refuse this option, the refusal to permit record linkage is itself a data point for analyzing patterns associated with concealment and dropout. Along the same lines, at each wave there should be included a query regarding the level of stress associated with completing the questionnaire.^183^ This may also help to better understand and estimate the effects of women subsequently dropping out.

Finally, it should be noted that it has already been shown that there may be significant differences in women’s experiences relative to different cultures and nationalites.^50^ Therefore, it is highly recommended that longitudinal studies to comprehensively investigate the intersections between mental and reproductive health should be funded in multiple countries.

### Data sharing for re-analyses should be rule rather than the exception

It is precisely because data can be selectively analyzed and interpreted to produce slanted results,^131–133^ that data should be made available for re-analyses by third parties.^276^ Data sharing also reduces the costs of research and magnifies the contribution volunteers make to science by making their non-identifying information accessible to more scientists, which presumably most volunteers would prefer as their participation is generally intended to help science in general, not specific research teams. Most importantly, data sharing enhances confidence in the reliability of research findings, especially when related to controversial issues. Unfortunately, though many publications and professional organizations encourage or require post-publication sharing of data, in practice many researchers across many disciplines evade data sharing.^277^

Support for data sharing, at least in theory, is found in the APA’s ethics rule 8.14, which states that following publication of their results, research psychologists should share the data for reanalysis by others.^278^ But this principle has been frequently ignored,^279–281^ especially in regard to abortion research. For example, the chair of the APA’s own TFMHA, Brenda Major, has repeatedly refused to allow data she collected on abortion patients to be subject to reanalysis by AMH proponents. She even refused to comply with a request for the data from the US Department of Health and Human Services, even though the study was funded by that agency.^140^

Such data hoarding undermines confidence not only in the published findings of a specific study but also diminishes the value of syntheses or reviews relying on those unverified findings.

Data sharing is especially important when the process of collecting data may be blocked by ideological litmus tests. For example, abortion providers are naturally unlikely to cooperate with studies initiated by AMH proponents who they perceive as opponents of their work. On the contrary, they have frequently cooperated with AMH minimalists—precisely because of their shared ideology. Implicit in granting that cooperation may be the expectation that pro-choice researchers will not report any findings that may contribute to anti-abortion rhetoric. Conversely, many post-abortion counseling programs may also limit their cooperation to AMH proponents whom they perceive as most accepting and supportive of the issues raised by their clientele.^88^

In both cases, the ideological alignments required to collect data may create biases in the design, analysis, and reporting of results. This does not mean that meaningful results cannot be obtained. But it does mean that such results should always be presumed to reflect sample and investigator biases until the findings have been confirmed in reanalyses conducted by investigators of all perspectives. It is only through equal access to the data that consensus will grow around results which survive reanalyses. It is also through this process that new research objectives will be better identified in response to these reanalyses.

### Responsiveness to requests for additional analyses

In many cases, legal restrictions (government or contractual) may bar the sharing of underlying data. In such cases, reasonable requests for additional information, tables, and reanalyses should be honored through personal communication, publication of a response, or, if a major reanalysis is required, in publication of a subsequent paper. Such cooperation is especially important in regard to data sets that have access restrictions, such as those collected by government agencies.

For example, the centralized medical records of Denmark have provided some of the best record linkage studies in the world. However, when it comes to mental health effects associated with abortion, there is strong evidence that significant findings are being suppressed for ideological reasons. The arguments and evidence for this assertion are given below.

In 2011, Munk-Olsen et al.^82^ published an analysis of Danish medical records to investigate first time psychiatric contact in the first year following a first abortion or first delivery. The analyses revealed that women who aborted had double the risk of psychiatric contact (OR = 2.18). But this finding was discounted by the finding that aborting women also had higher rates of outpatient psychiatric contact in the 9 months prior to their abortions (including the time they were pregnant) compared to the 9 months prior to a live birth. Munk-Olsen later conceded that this mixture of pre-conception time and pregnancy time created a baseline that “may not be directly compatible.”^227^ But this was just one of many major weaknesses in the design and reporting of this highly criticized study.^282^

Another methodological problem was the decision to include women who had one or more abortions prior to their first delivery into the delivery group. This decision is especially problematic since a history of abortion is significantly associated with higher rates of mental illness during and after subsequent pregnancies.^78,80,99,170,197,217^ Notably, when Munk-Olsen was asked to provide a simple count of the number of women in her analyses who had both abortions and deliveries and the percentage of those who had psychiatric contact, she refused this and all other requests for more details.^227^

Before examining the inconsistencies revealed in subsequent Munk-Olsen et al.^82^ studies, it is relevant to compare her abortion study to three very similar record linkage studies conducted by AMH proponents conducted a decade earlier. These prior studies examined the differences between abortion and delivery in regard to inpatient psychiatric treatments,^89^ outpatient psychiatric treatments,^97^ and sleep disorders.^87^ The designs of those studies were superior to Munk-Olsen’s in several respects: (a) in each case, controls for prior psychiatric inpatient treatment were employed for a longer period of time, a 12- to 18-month period prior to the estimated *date of conception* for each woman; (b) there was complete segregation of women relative to exposure to abortion; (c) mental health outcomes were reported showing variations relative to different age groups; and (d) results were shown over multiple time periods: 0–90 days, 0–180 days, first year, second year, third year, fourth year, and 0–4 years.

Normally, one would expect Munk-Olsen to have at least replicated, if not improved on, the methodology employed in these prior record linkage studies. Instead, the methodological choices she made severely narrowed the range of her investigation. Studies that are narrowly drawn can only support narrow conclusions. This is especially true since Munk-Olsen also excluded any analyses of the effects of multiple abortions, which are known to be associated with even higher rates of negative reactions^94,112^ and also make up the majority of all abortions being performed.^64^

Concerns about selective reporting are heighted by the fact that Munk-Olsen subsequently published numerous studies on mental health associated with childbirth in which, once again, she refused requests to supply data for findings associated with abortion. For example, using the same data set, Munk-Olsen published findings that reported

1. Psychiatric treatment following delivery was associated with a fourfold increased risk of a diagnosis of bipolar disorders within the next 15 years;^283^
2. Rates of antidepressant use and mental health treatments 12 months prior to childbirth and 12 months after;^208^
3. Elevated rates of psychiatric disorders following miscarriage or stillbirth;^217^
4. Rates of postpartum depression following delivery of IVF pregnancies;^284^
5. Rates of primary care treatments before, during, and after pregnancies in which women experienced postpartum psychiatric episodes;^210^
6. Average monthly rates of psychological treatment and prescriptions before and after childbirth.^209^

In each of these cases, her analyses and conclusions were flawed by the failure to address the effects of prior fetal loss, which are known to increase the risk of psychiatric disorders during and after subsequent pregnancies.^78,170,212,225,285,286^

While in most cases she simply omitted abortion history from her analyses,^208–210,283^ in two cases she used abortion history as a control variable^217,284^ but omitted any statistics showing how this control affected the results. Clearly, the only reason to use abortion history as a control is if it has a significant independent effect on mental health outcomes.

The possibility that Munk-Olsen simply overlooked these opportunities to report on effects associated with abortion is disproven by the fact that in each case Munk-Olsen rejected both published^141,227^ and unpublished requests for details relative to the effects of abortion on the outcomes studied. Even a request for a simple count of the number of women exposed to abortion in each of Munk-Olsen’s comparison groups was refused.^141^

All of the above factors give credence to the concern that there is a selective withholding of results, by Munk-Olsen and other AMH minimalists. Moreover, given the evidence that abortion and miscarriage impacts mental health during subsequent pregnancies,^78,80,99,170,197,203,212–221^ it is clear that every study examining the intersection between mental and reproductive health may be misleading if it fails to include analyses associated with pregnancy loss. Without such analyses, effects associated with pregnancy loss may be wrongly attributed to childbirth.

For example, there is strong evidence from both record linkage^89,97^ and case-matched studies^287^ that a history of abortion is associated with a threefold increase in bipolar disorder. Therefore, Munk-Olsen et al.’s^283^ decision to exclude analyses related to fetal loss from her study of bipolar disorders following postpartum depression severely undermines her conclusion that this negative outcome is due to childbirth alone precisely because she chose to ignore, or at least not publish, findings associated with fetal loss.

The combination of Munk-Olsen’s failure to publish these results without being asked, combined with her refusal to respond to requests for reanalysis,^141,227^ strongly suggests a pattern of selective reporting and obfuscation. If the additional analyses requested actually supported her previous assertion that prior mental health fully explains the higher rates of mental illness seen among women who have aborted^82,107^ it seems clear that she should be rushing to publish these requested analyses precisely to silence skeptics.

In short, whenever either AMH minimalists or AMH proponents refuse to respond to queries for reanalyses of published findings, they are increasing distrust and weakening the credibility of all conclusions based on their previously published research. This creates real obstacles in the advance of evidence-based medicine, informed consent practices, and ultimately in the medical care of women. The advance of scientific investigations into reproductive mental health can only be enhanced by generously responding to requests for details and re-analyses that clarify the interpretation of published findings.

### Recommendations for editors and peer reviewers

As previously discussed, there is strong evidence that individual biases may unfairly bias editors and reviewers against findings that challenge their preconceived notions.^118–123^ Biases against “conservative” viewpoints, which may attach to the AMH controversy, are especially common.^125–128,130^

Editors should guard against this bias by seeking a mix of peer reviewers, including both AMH minimalists and AMH proponents. For reasons discussed previously, while recognizing that every study in this area will have methodological weaknesses and that no sample can be perfect, editors should be blind to the results and focus their evaluation of peer review comments on the appropriateness and adequacy of the methodology and study sample. Editors should be alert to criticisms that appear to reflect a reviewer’s bias against results which support an undesired conclusion, especially when the methodology employed is comparable to studies that would be accepted for publication in any other field of research.

A good test of bias is to simply imagine that the results were flipped,^123^ with the ORs showing benefits to abortion compared to delivering an unwanted pregnancy, for example. If the reviewer’s or editors reactions to the paper would most likely have been in the opposite direction, that reaction is obviously driven by a bias for preferred results.

Editors and peer reviewers should also strive to ensure that all studies relating to the intersection of mental and reproductive health include, whenever possible, analyses that delineate findings relative to exposure to all prior pregnancy outcomes, including both natural pregnancy losses and induced abortions.^141,227^ This is important for several reasons. First, there is consensus even among AMH minimalists that better data are needed on the effects of pregnancy loss on mental health.^4,274^ Second, there is clear and convincing evidence that exposure to pregnancy losses (both natural and induced) may have a significant impact on women’s health during and after subsequent pregnancies and at other times in women’s lives.^80,88,94,99,112,170,212,285^

When data on abortion and miscarriage history are available, but not included in published findings, this raises concerns about concealment of findings that the authors may be afraid will bolster the position of their ideological rivals.^141,227^ Alert reviewers and editors should routinely ask researchers to include in their tables of results analyses relevant to the number of exposures to abortion and natural pregnancy losses. Without such requests (a) the literature will continue to be deprived of meaningful data and (b) selective reporting may falsely attribute negative mental health issues to childbirth.

## Limitations

The purpose of this review of the medical literature on AMH was to examine the areas of agreement and disagreement, the reasons for disagreement, and the opportunities for improved research and collaboration. The method I used began with a review of reviews published since 2005^4–10,12–19,21,22^ and an examination of the studies cited in these reviews.

Given the difficulties previously discussed in conducting any conclusive studies, the breadth of issues examined in this review, and the range of theories and opinions of the authors of the reviews and studies examined, it is out of the scope of this, or any, review to fully address every view or concern. With that limitation in mind, however, this review does catalog a broader range of relevant issues than any previous reviews. In doing so, this review does not offer the last word on the AMH controversy. Instead, it seeks to expand and continue the conversation, inviting more detailed responses, criticism, and elaboration regarding the issues identified herein.

## Conclusion

While there will continue to be differences of opinion between AMH minimalists and AMH proponents, there is sufficient common ground upon which to build future efforts to improve research and meaningful re-analyses. Common ground exists regarding the very basic fact that at least some women do have significant mental health issues that are caused, triggered, aggravated, or complicated by their abortion experience. In many cases, this may be due to feeling pressured into an abortion or choosing an abortion without sufficient attention to maternal desires or moral beliefs that may make it difficult to reconcile one’s choice with one’s self-identity.

There is also common ground regarding the fact that risk factors identifying women who are at greater risk, including a history of prior mental illness, can be used to identify women who may benefit from more pre-abortion and post-abortion counseling. Additional research regarding risk factors, and indicators identifying when abortion may be most likely to produce the benefits sought by women without negative consequences, can and should be conducted through major longitudinal prospective studies.

Finally, there is common ground on the need for better research. That fact alone is a strong argument for mixed research teams, collaboration in the design of longitudinal studies available for analysis by any researcher (without ideological screenings), data sharing and more responsive cooperation in responding to requests for reanalysis. All of these steps will help to provide healthcare workers with more accurate information for screening, risk–benefits assessments, and for offering better care and information to women both before and after abortion and other reproductive events.

## References

[1] Wilmoth GH. Abortion, public health policy, and informed consent legislation. J Soc Issues 1992; 48(3): 1–17. 11656501 10.1111/j.1540-4560.1992.tb00895.x

[2] Painter J Ashe T. Cross-national comparison of the presence of climate scepticism in the print media in six countries, 2007–10. Environ Res Lett 2012; 7(4): 044005.

[3] MacNair RM. Introduction: understanding perspectives. In: MacNair RM (ed.) Peace psychology perspectives on abortion. Kansas City, MO: Feminism & Nonviolence Studies Association, 2016, p. 316.

[4] Major B Appelbaum M Beckman L et al Report of the APA Task Force on mental health and abortion. Washington, DC: American Psychological Association, 2008, 105 pp, http://www.apa.org/pi/women/programs/abortion/mental-health.pdf

[5] National Collaborating Centre for Mental Health. Induced abortion and mental health: a systematic review of the mental health outcomes of induced abortion, including their prevalence and associated factors. London: Academy of Medical Royal Colleges, 2011, http://www.aomrc.org.uk/wp-content/uploads/2016/05/Induced_Abortion_Mental_Health_1211.pdf

[6] Charles VE Polis CB Sridhara SK et al Abortion and long-term mental health outcomes: a systematic review of the evidence. Contraception 2008; 78: 436–450. 19014789 10.1016/j.contraception.2008.07.005

[7] Major B Appelbaum M Beckman L et al Abortion and mental health: evaluating the evidence. Am Psychol 2009; 64(9): 863–890. 19968372 10.1037/a0017497

[8] Steinberg JR. Later abortions and mental health: psychological experiences of women having later abortions—a critical review of research. Womens Health Issues 2011; 21(3): S44–S48. 21530839 10.1016/j.whi.2011.02.002

[9] Hall M Chappell LC Parnell BL et al Associations between intimate partner violence and termination of pregnancy: a systematic review and meta-analysis. PLoS Med 2014; 11(1): e1001581. 24409101 10.1371/journal.pmed.1001581 PMC3883805

[10] Horvath S Schreiber CA. Unintended pregnancy, induced abortion, and mental health. Curr Psychiatry Rep 2017; 19(11): 77. 28905259 10.1007/s11920-017-0832-4

[11] Thorp JM Hartmann KE Shadigian E. Long-term physical and psychological health consequences of induced abortion: review of the evidence. Obstet Gynecol Surv 2003; 58(1): 67–79. 12544786 10.1097/00006254-200301000-00023

[12] Casey P Oates M Jones I et al Invited commentaries on… Abortion and mental health disorders. Br J Psychiatry 2008; 193(6): 452–454. 19043145 10.1192/bjp.bp.108.059550

[13] Coleman PK. Induced abortion and increased risk of substance abuse: a review of the evidence. Curr Womens Health Rev 2005; 1(1): 21–34

[14] Coleman PK Reardon DC Strahan T et al The psychology of abortion: a review and suggestions for future research. Psychol Health 2005; 20: 237–271.

[15] Coleman PK. Abortion and mental health: quantitative synthesis and analysis of research published 1995-2009. Br J Psychiatry 2011; 199: 180–186. 21881096 10.1192/bjp.bp.110.077230

[16] Bellieni CV Buonocore G. Abortion and subsequent mental health: review of the literature. Psychiatry Clin Neurosci 2013; 67(5): 301–310. 23859662 10.1111/pcn.12067

[17] Fergusson DM Horwood LJ Boden JM. Does abortion reduce the mental health risks of unwanted or unintended pregnancy? A re-appraisal of the evidence. Aust N Z J Psychiatry 2013; 47(9): 819–827. 23553240 10.1177/0004867413484597

[18] Gurpegui M Jurado D. Psychiatric complications of abortion. Cuad Bioét 2009; 20(70): 381–392. 19799479

[19] Ney PG. A common sense scientific critique of the NCCMH and Royal College of Psychiatry Review. Webmed Cent 2013; 4(10): 1–13.

[20] Turk AM How we got there from here: transforming the debate over abortion. In: MacNair RM (ed.) Peace psychology perspectives on abortion. Kansas City, MO: Feminism & Nonviolence Studies Association, 2016, p. 312.

[21] Daugirdaitė V van den Akker O Purewal S. Posttraumatic stress and posttraumatic stress disorder after termination of pregnancy and reproductive loss: a systematic review. J Pregnancy 2015; 2015: 646345 (14 pp.). 10.1155/2015/646345 PMC4334933 25734016

[22] Hanschmidt F Linde K Hilbert A et al Abortion stigma: a systematic review. Perspect Sex Reprod Health 2016; 48(4): 169–177. 27037848 10.1363/48e8516

[23] Coleman PK Reardon DC Lee MB. Women’s preferences for information and complication seriousness ratings related to elective medical procedures. J Med Ethics 2006; 32(8): 435–438. 16877620 10.1136/jme.2005.014274 PMC2563388

[24] Major B Cozzarelli C. Psychosocial predictors of adjustment to abortion. J Soc Issues 1992; 48(3): 121–142.

[25] Reardon DC. Abortion decisions and the duty to screen: clinical, ethical, and legal implications of predictive risk factors of post-abortion maladjustment. J Contemp Health Law Policy 2003; 20(1): 33–114. 15067928

[26] Royal College of Psychiatrists. Position statement of women’s mental health in relation to induced abortion. London, 2008, https://www.wthrockmorton.com/2008/08/20/royal-college-of-psychiatrists-statement-on-abortion-and-mental-health/

[27] Shuping M. Wantedness & coercion: key factors in understanding women’s mental health after abortion. Assoc Interdiscip Res Values Soc Chang Res Bull 2011; 23(2): 8.

[28] Shusterman L. The psychosocial factors of the abortion experience: a critical review. Psychol Women Q 1976; 1: 79–106. 12334843 10.1111/j.1471-6402.1976.tb00810.x

[29] Shusterman L. Predicting the psychological consequences of abortion. Soc Sci Med Med Psychol Med Sociol 1979; 13: 683–689. 10.1016/0271-7123(79)90113-5 538481

[30] Steinberg JR McCulloch CE Adler NE. Abortion and mental health: findings from the national comorbidity survey-replication. Obstet Gynecol 2014; 123(2 Pt 1): 263–270. 24402590 10.1097/AOG.0000000000000092 PMC3929105

[31] Lehmann C. Psychiatrists can play critical role in pregnancy decisions. Psychiatr News 2003; 38(13): 8–43.

[32] Athanasiou R Oppel W Michelson L et al Psychiatric sequelae to term birth and induced early and late abortion: a longitudinal study. Fam Plann Perspect 1973; 5(4): 227–231. 4156672

[33] Belsey EM Greer HS Lal S et al Predictive factors in emotional response to abortion: King’s termination study —IV. Soc Sci Med 1977; 11(2): 71–82. 594780 10.1016/0037-7856(77)90002-6

[34] Casey PR. Abortion among young women and subsequent life outcomes. Best Pract Res Cl Ob 2010; 24(4): 491–502. 10.1016/j.bpobgyn.2010.02.007 20303829

[35] Coleman PK. Women at risk for post-abortion mental health problems and abortion associated relationship challenges. Post-abortion trauma: possible psychological and existential aftermaths. Rome: Pontifical Academy for Life, 2014, pp. 147–210, http://www.academiavita.org/_pdf/documents/pav/post_abortion_trauma.pdf

[36] Lask B. Short term psychiatric sequelae to therapeutic termination of pregnancy. Br J Psychiatry 1975; 126(2): 173–177. 1131468 10.1192/bjp.126.2.173

[37] Lemkau J. Emotional sequelae of abortion: implications for clinical practice. Psychol Women Q 1988; 12: 461–472. 12283699 10.1111/j.1471-6402.1988.tb00978.x

[38] Miller W. An empirical study of the psychological antecedents and consequences of induced abortion. J Soc Issues 1992; 48(3): 67–93.

[39] Major B Cozzarelli C Cooper ML et al Psychological responses of women after first-trimester abortion. Arch Gen Psychiatry 2000; 57(8): 777–784. 10920466 10.1001/archpsyc.57.8.777

[40] Stotland NL. Abortion: social context, psychodynamic implications. Am J Psychiatry 1998; 155(7): 964–967. 9659865 10.1176/ajp.155.7.964

[41] Torre-Bueno A. Peace after abortion. San Diego, CA: Pimpernel Press, 1997.

[42] Johnson TM. Bringing abortion aftercare into the 21st century. Counseling Today, 2013, http://ct.counseling.org/2013/01/bringing-abortion-aftercare-into-the-21st-century/

[43] De Puy C Dovitch D The healing choice: your guide to emotional recovery after an abortion. New York: Simon & Schuster, 1997.

[44] Goldstein D. The abortion counseling conundrum. The American Prospect, 2008, http://prospect.org/article/abortion-counseling-conundrum

[45] Burke T Reardon DC. Forbidden grief: the unspoken pain of abortion. Springfield, IL: Acorn Books, 2007, 334 pp.

[46] Layer SD. Postabortion grief: evaluating the possible efficacy of a spiritual group intervention. Res Soc Work Pract 2004; 14(5): 344–350.

[47] Ney PG Wickett AR. Mental health and abortion: review and analysis. Psychiatr J Univ Ott 1989; 14(4): 506–516. 2682716

[48] Zimmerman MK. Passage through abortion: the personal and social reality of women’s experiences. New York: Praeger Publishers, 1977, 238 pp.

[49] Sihvo S Bajos N Ducot B et al Women’s life cycle and abortion decision in unintended pregnancies. J Epidemiol Community Health 2003; 57(8): 601–605. 12883066 10.1136/jech.57.8.601 PMC1732542

[50] Rue VM Coleman PK Rue JJ et al Induced abortion and traumatic stress: a preliminary comparison of American and Russian women. Med Sci Monit 2004; 10(10): SR5–SR16. 15448616

[51] Major B Mueller P Hildebrandt K. Attributions, expectations, and coping with abortion. J Pers Soc Psychol 1985; 48(3): 585–599. 3989663 10.1037//0022-3514.48.3.585

[52] Smetana J. Reasoning in the personal and moral domains: adolescent and young adult women’s decision-making regarding abortion. J Appl Dev Psychol 1981; 2: 211–226.

[53] Major B Gramzow RH. Abortion as stigma: cognitive and emotional implications of concealment. J Pers Soc Psychol 1999; 77(4): 735–745. 10531670 10.1037//0022-3514.77.4.735

[54] Cozzarelli C Major B Karrasch A. Women’s experiences of and reactions to antiabortion picketing. Basic Appl Soc 2000; 22: 265–275.

[55] Major B Zubek JM Cooper ML et al Mixed messages: implications of social conflict and social support within close relationships for adjustment to a stressful life event. J Pers Soc Psychol 1997; 72(6): 1349–1363. 9177021 10.1037//0022-3514.72.6.1349

[56] Major B Cozzarelli C Sciacchitano AM et al Perceived social support, self-efficacy, and adjustment to abortion. J Pers Soc Psychol 1990; 59(3): 452–463. 2231279 10.1037//0022-3514.59.3.452

[57] Steinberg JR Finer LB. Examining the association of abortion history and current mental health: a reanalysis of the National Comorbidity Survey using a common-risk-factors model. Soc Sci Med 2011; 72(1): 72–82. 21122964 10.1016/j.socscimed.2010.10.006

[58] Emmerik A van Kamphuis J. Prevalence and prediction of re-experiencing and avoidance after elective surgical abortion: a prospective study. Clin Psychol Psychother 2008; 15: 378–385. 19115456 10.1002/cpp.586

[59] Broen AN Moum T Bödtker AS et al Psychological impact on women of miscarriage versus induced abortion: a 2-year follow-up study. Psychosom Med 2004; 66(2): 265–271. 15039513 10.1097/01.psy.0000118028.32507.9d

[60] Van Rooyenm M Smith S The prevalence of post-abortion syndrome in patients presenting at Kalafong hospital’s family medicine clinic after having a termination of pregnancy. S Afr Fam Pr 2004; 46(5): 21–24.

[61] Husfeldt C Hansen SK Lyngberg A et al Ambivalence among women applying for abortion. Acta Obstet Gynecol Scand 1995; 74(10): 813–817. 8533566 10.3109/00016349509021203

[62] Kero A Högberg U Jacobsson L et al Legal abortion: a painful necessity. Soc Sci Med 2001; 53(11): 1481–1490. 11710423 10.1016/s0277-9536(00)00436-6

[63] Cohan CL Dunkel-schetter C Lydon J. Pregnancy decision making: predictors of early stress and adjustment. Psychol Women Q 1993; 17(2): 223–239. 12345377 10.1111/j.1471-6402.1993.tb00446.x

[64] Jones R Singh S Finer L et al Repeat abortion in the United States. Occasional report no. 29. New York, 2006, http://www.popline.org/node/563305

[65] Pazol K Creanga AA Burley KD et al; Centers for Disease Control and Prevention (CDC). Abortion surveillance—United States, 2010. MMWR Surveill Summ 2013; 62(8): 1–44. 24280963

[66] Baker A Beresford T. Informed consent, patient education and counseling. In: Paul M Lichtenberg E Borgatta L et al (eds) Management of unintended and abnormal pregnancy: comprehensive abortion care. Chichester: Wiley-Blackwell, 2009, https://books.google.com/books?hl=en&lr=&id=iK7xrRr2p9sC&oi=fnd&pg=PA27&dq=Management+of+Unintended+and+Abnormal+Pregnancy:+Comprehensive+Abortion+Care&ots=S1VJfHUdVD&sig=C5Yqy9-jPROhk2wPikMS87GTWTo#v=onepage&q=ManagementofUnintendedandAbnormalPregna

[67] Gissler M Karalis E Ulander VM. Decreased suicide rate after induced abortion, after the Current Care Guidelines in Finland 1987–2012. Scand J Public Health 2014; 43(1): 99–101. 25420710 10.1177/1403494814560844

[68] Upadhyay UD Cockrill K Freedman LR. Informing abortion counseling: an examination of evidence-based practices used in emotional care for other stigmatized and sensitive health issues. Patient Educ Couns 2010; 81(3): 415–421. 20926226 10.1016/j.pec.2010.08.026

[69] Schmiege S Russo NF. Depression and unwanted first pregnancy: longitudinal cohort study. BMJ 2005; 331(7528): 1303. 10.1136/bmj.38623.532384.55 PMC1298850 16257993

[70] Reardon DC. Missed opportunities and overstated results in anxiety and quality of life study following termination of pregnancy. Acta Obstet Gynecol Scand 2016; 96: 382. 27897315 10.1111/aogs.13053

[71] Cougle JR Reardon DC Coleman PK. Depression associated with abortion and childbirth: a long-term analysis of the NLSY cohort. Med Sci Monit 2003; 9(4): CR105–CR112. 12709667

[72] Cougle JR Reardon DC Coleman PK. Generalized anxiety following unintended pregnancies resolved through childbirth and abortion: a cohort study of the 1995 National Survey of Family Growth. J Anxiety Disord 2005; 19(1): 137–142. 15488373 10.1016/j.janxdis.2003.12.003

[73] David HP. Post-abortion and post-partum psychiatric hospitalization. Ciba Found Symp 1985; 115: 150–164. 3849411 10.1002/9780470720967.ch12

[74] Dingle K Alati R Clavarino A et al Pregnancy loss and psychiatric disorders in young women: an Australian birth cohort study. Br J Psychiatry 2008; 193(6): 455–460. 19043146 10.1192/bjp.bp.108.055079

[75] Fergusson DM Horwood LJ Boden JM. Abortion and mental health disorders: evidence from a 30-year longitudinal study. Br J Psychiatry 2008; 193(6): 444–451. 19043144 10.1192/bjp.bp.108.056499

[76] Gilchrist AC Hannaford PC Frank P et al Termination of pregnancy and psychiatric morbidity. Br J Psychiatry 1995; 167(2): 243–248. 7582677 10.1192/bjp.167.2.243

[77] Gissler M Hemminki E Lönnqvist J et al Suicides after pregnancy in Finland, 1987-94: register linkage study. BMJ 1996; 313(7070): 1431–1434. 8973229 10.1136/bmj.313.7070.1431 PMC2352979

[78] Gong X Hao J Tao F et al Pregnancy loss and anxiety and depression during subsequent pregnancies: data from the C-ABC study. Eur J Obstet Gynecol Reprod Biol 2013; 166(1): 30–36. 23146315 10.1016/j.ejogrb.2012.09.024

[79] Luo M Jiang X Wang Y et al Association between induced abortion and suicidal ideation among unmarried female migrant workers in three metropolitan cities in China: a cross-sectional study. BMC Public Health 2018; 18(1): 625. 29764402 10.1186/s12889-018-5527-1 PMC5952593

[80] Meltzer-Brody S Maegbaek ML Medland SE et al Obstetrical, pregnancy and socio-economic predictors for new-onset severe postpartum psychiatric disorders in primiparous women. Psychol Med 2017; 47: 1427–1441. 28112056 10.1017/S0033291716003020 PMC5429203

[81] Morgan CL Evans M Peters JR. Suicides after pregnancy. Mental health may deteriorate as a direct effect of induced abortion. BMJ 1997; 314(7084): 902. PMC2126260 9093118

[82] Munk-Olsen T Laursen TM Pedersen CB et al Induced first-trimester abortion and risk of mental disorder. N Engl J Med 2011; 364(4): 332–339. 21268725 10.1056/NEJMoa0905882

[83] Østbye T Wenghofer EF Woodward CA et al Health services utilization after induced abortions in Ontario: a comparison between community clinics and hospitals. Am J Med Qual 2001; 16(3): 99–106. 11392176 10.1177/106286060101600305

[84] Pedersen W. Childbirth, abortion and subsequent substance use in young women: a population-based longitudinal study. Addiction 2007; 102(12): 1971–1978. 18031432 10.1111/j.1360-0443.2007.02040.x

[85] Pedersen W. Abortion and depression: a population-based longitudinal study of young women. Scand J Public Health 2008; 36(4): 424–428. 18539697 10.1177/1403494807088449

[86] Reardon DC Cougle JR. Depression and unintended pregnancy in the National Longitudinal Survey of Youth: a cohort study. BMJ 2002; 324(7330): 151–152. 11799033 10.1136/bmj.324.7330.151 PMC64517

[87] Reardon DC Coleman PK. Relative treatment rates for sleep disorders and sleep disturbances following abortion and childbirth: a prospective record-based study. Sleep 2006; 29(1): 105–106. 16453987 10.1093/sleep/29.1.105

[88] Reardon DC Ney PG Scheuren F et al Deaths associated with pregnancy outcome: a record linkage study of low income women. South Med J 2002; 95(8): 834–841. 12190217

[89] Reardon DC Cougle JR Rue VM et al Psychiatric admissions of low-income women following abortion and childbirth. CMAJ 2003; 168(10): 1253–1256. 12743066 PMC154179

[90] Reardon DC Coleman PK Cougle JR. Substance use associated with unintended pregnancy outcomes in the National Longitudinal Survey of Youth. Am J Drug Alcohol Abuse 2004; 30(2): 369–383. 15230081 10.1081/ada-120037383

[91] Rees D Sabia J. The relationship between abortion and depression: new evidence from the fragile families and child wellbeing study. Med Sci Monit 2007; 13(10): CR430–CR436. 17901849 10.12659/msm.502357

[92] Steinberg JR Russo NF. Abortion and anxiety: what’s the relationship? Soc Sci Med 2008; 67(2): 238–252. 18468755 10.1016/j.socscimed.2008.03.033

[93] Steinberg JR Laursen TM Adler NE et al Examining the association of antidepressant prescriptions with first abortion and first childbirth. JAMA Psychiatry 2018; 75: 828–834. 29847626 10.1001/jamapsychiatry.2018.0849 PMC6143090

[94] Sullins DP. Abortion, substance abuse and mental health in early adulthood: thirteen-year longitudinal evidence from the United States. SAGE Open Med 2016; 4: 2050312116665997. 27781096 10.1177/2050312116665997 PMC5066584

[95] Taft AJ Watson LF. Depression and termination of pregnancy (induced abortion) in a national cohort of young Australian women: the confounding effect of women’s experience of violence. BMC Public Health 2008; 8: 75. 18302736 10.1186/1471-2458-8-75 PMC2278138

[96] van Ditzhuijzen J ten Have M de Graaf R et al Incidence and recurrence of common mental disorders after abortion: results from a prospective cohort study. J Psychiatr Res 2017; 84: 200–206. 27760409 10.1016/j.jpsychires.2016.10.006

[97] Coleman PK Reardon DC Rue VM et al State-funded abortions versus deliveries: a comparison of outpatient mental health claims over 4 years. Am J Orthopsychiatry 2002; 72(1): 141–152. 14964603 10.1037/0002-9432.72.1.1410155

[98] Coleman PKP Resolution of unwanted pregnancy during adolescence through abortion versus childbirth: individual and family predictors and psychological consequences. J Youth Adolesc 2006; 35(6): 903–911.

[99] Coleman PK Reardon DC Rue VM et al A history of induced abortion in relation to substance abuse during subsequent pregnancies carried to term. Am J Obstet Gynecol 2002; 187(6): 1673–1678. 12501082 10.1067/mob.2002.127602

[100] Coleman PK Reardon DC Cougle JR. Substance use among pregnant women in the context of previous reproductive loss and desire for current pregnancy. Br J Health Psychol 2005; 10(Pt 2): 255–268. 15969853 10.1348/135910705X25499

[101] Coleman PK Coyle CT Shuping M et al Corrigendum to “induced abortion and anxiety, mood, and substance abuse disorders: isolating the effects of abortion in the national comorbidity survey” [Journal of Psychiatric Research 2009;43:770–776]. J Psychiatr Res 2011; 45(8): 1133–1134. 10.1016/j.jpsychires.2008.10.009 19046750

[102] Coleman PK Maxey CD Spence M et al Predictors and correlates of abortion in the fragile families and well-being study: paternal behavior, substance use, and partner violence. Int J Ment Health Addict 2009; 7(3): 405–422.

[103] Mota NP Burnett M Sareen J. Associations between abortion, mental disorders, and suicidal behaviour in a nationally representative sample. Can J Psychiatry 2010; 55(4): 239–247. 20416147 10.1177/070674371005500407

[104] Steinberg JR Trussell J Hall KS et al Fatal flaws in a recent meta-analysis on abortion and mental health. Contraception 2012; 86(5): 430–437. 22579105 10.1016/j.contraception.2012.03.012 PMC3646711

[105] Major B. Psychological implications of abortion—highly charged and rife with misleading research. CMAJ 2003; 168(10): 1257–1258. 12743067 PMC154180

[106] Steinberg JR Tschann JM. Childhood adversities and subsequent risk of one or multiple abortions. Soc Sci Med 2013; 81: 53–59. 23312795 10.1016/j.socscimed.2012.11.011 PMC3699177

[107] Munk-Olsen T Laursen TM Pedersen CB et al First-time first-trimester induced abortion and risk of readmission to a psychiatric hospital in women with a history of treated mental disorder. Arch Gen Psychiatry 2012; 69(2): 159–165. 22310504 10.1001/archgenpsychiatry.2011.153

[108] Coyle C. Intimate partner violence. In: MacNair RM (ed.) Peace psychology perspectives on abortion. Kansas City, MO: Feminism & Nonviolence Studies Association, 2016.

[109] Coyle CT Shuping MW Speckhard A et al The relationship of abortion and violence against women: violence prevention strategies and research needs. Issues Law Med 2015; 30(2): 111–127. 26710370

[110] Russo NF Denious JE. Violence in the lives of women having abortions: implications for practice and public policy. Prof Psychol Res Pract 2001; 32(2): 142–150.

[111] Scott KM Lim C Al-Hamzawi A et al Association of mental disorders with subsequent chronic physical conditions: world mental health surveys from 17 countries. JAMA Psychiatry 2016; 73(2): 150–158. 26719969 10.1001/jamapsychiatry.2015.2688 PMC5333921

[112] Coleman PK Reardon DC Calhoun BC. Reproductive history patterns and long-term mortality rates: a Danish, population-based record linkage study. Eur J Public Health 2013; 23(4): 569–574. 22954474 10.1093/eurpub/cks107

[113] Reardon DC Coleman PK. Short and long term mortality rates associated with first pregnancy outcome: population register based study for Denmark 1980-2004. Med Sci Monit 2012; 18(9): PH71–PH76. 22936199 10.12659/MSM.883338 PMC3560645

[114] APA Press Room. APA Task Force finds single abortion not a threat to women’s mental health. American Psychological Association Press Releases, 2008, http://www.apa.org/news/press/releases/2008/08/single-abortion.aspx

[115] Jones R Finer L Singh S. Characteristics of US abortion patients, 2008. New York: Guttmacher Institute, 2010, http://nyfamilylife.org/wp-content/uploads/2013/11/US-Abortion-Patients.pdf

[116] Jones R Kost K Singh S et al Trends in abortion in the United States. Clin Obstet Gynecol 2009; 52(2): 119–129. 19407518 10.1097/GRF.0b013e3181a2af8f

[117] Finer LB Frohwirth LF Dauphinee LA et al.Reasons U.S. women have abortions: quantitative and qualitative perspectives. Perspect Sex Reprod Health 2005; 37(3): 110–118. 16150658 10.1363/psrh.37.110.05

[118] Biggs M. The impact of peer review on intellectual freedom. Libr Trends 1990; 39: 145–167.

[119] Abramowitz SI Gomes B Abramowitz C V. Publish or politic: referee bias in manuscript review. J Appl Soc Psychol 1975; 5(3): 187–200.

[120] Armstrong JS. Peer review for journals: evidence on quality control, fairness, and innovation. Sci Eng Ethics 1997; 3(1): 63–84.

[121] Peters D Ceci S. Peer-review practices of psychological journals: the fate of published articles, submitted again. Behav Brain Sci 1982; 5: 187–195.

[122] Koehler JJ. The influence of prior beliefs on scientific judgments of evidence quality. Organ Behav Hum Decis Process 1993; 56(1): 28–55.

[123] Mahoney MJ. Publication prejudices: an experimental study of confirmatory bias in the peer review system. Cognit Ther Res 1977; 1(2): 161–175.

[124] Lord CG Ross L Lepper MR. Biased assimilation and attitude polarization: the effects of prior theories on subsequently considered evidence. J Pers Soc Psychol 1979; 37(11): 2098–2109.

[125] Goodstein LD Brazis KL. Psychology of scientist: XXX. Credibility of psychologists: an empirical study. Psychol Rep 1970; 27(3): 835–838.

[126] Wright RH Cummings NA (eds). Destructive trends in mental health: the well intentioned path to harm. New York, NY: Routledge, 2005, 384 pp.

[127] Konnikova M. Is social psychology biased against Republicans? The New Yorker, 2014, http://www.newyorker.com/science/maria-konnikova/social-psychology-biased-republicans

[128] Duarte JL Crawford JT Stern C et al Political diversity will improve social psychological science. Behav Brain Sci 2015; 38: e130. 25036715 10.1017/S0140525X14000430

[129] Tappin BM McKay RT. The illusion of moral superiority. Soc Psychol Personal Sci 2017; 8: 623–631. 29081899 10.1177/1948550616673878 PMC5641986

[130] Inbar Y Lammers J. Political diversity in social and personality psychology. Perspect Psychol Sci 2012; 7(5): 496–503. 26168506 10.1177/1745691612448792

[131] Open Science Collaboration. Estimating the reproducibility of psychological science. Science 2015; 349(6251): aac4716. 10.1126/science.aac4716 26315443

[132] Ioannidis JPA Why most published research findings are false. PLoS Med 2005; 2(8): e124. 16060722 10.1371/journal.pmed.0020124 PMC1182327

[133] Simmons JP Nelson LD Simonsohn U. False-positive psychology: undisclosed flexibility in data collection and analysis allows presenting anything as significant. Psychol Sci 2011; 22(11): 1359–1366. 22006061 10.1177/0956797611417632

[134] Tierney J. Social psychologists detect liberal bias within. The New York Times, 7 2 2011, http://www.nytimes.com/2011/02/08/science/08tier.html?_r=1

[135] American Psychological Association. Council policy manual: abortion resolutions. American Psychological Association, 1969, p. 1, http://www.apa.org/about/policy/abortion.aspx

[136] Coleman PK Deriving sensible conclusion from the scientific literature on abortion and women’s mental health. In: MacNair RM (ed.) Peace psychology perspectives on abortion. Kansas City, MO: Feminism & Nonviolence Studies Association, 2016, pp. 74–93.

[137] Hill R. Abortion researcher confounded by study. New Zealand Herald, 1 5 2006, http://www.nzherald.co.nz/nz/news/article.cfm?c_id=1&objectid=10362476

[138] Fergusson DM Horwood LJ Ridder EM. Abortion in young women and subsequent mental health. J Child Psychol Psychiatry Allied Discip 2006; 47(1): 16–24. 10.1111/j.1469-7610.2005.01538.x 16405636

[139] Speckhard A. Post abortion distress—the politically incorrect trauma. Anne Speckhard, Wordpress.com, 2013, https://annespeckhard.wordpress.com/2013/05/14/post-abortion-distress-the-politically-incorrect-trauma/

[140] Sobie A. Lead author refuses to release abortion data collected under federal grant. AfterAbortion.org, 13 8 2008, http://afterabortion.org/2008/chair-of-apa-abortion-report-task-force-violates-apa-ethics-rules/

[141] Reardon DC. Postpartum mental health study flawed by fetal loss omission. Scand J Prim Health Care 2015; 33(4): 318–319. 26683289 10.3109/02813432.2015.1111710 PMC4750743

[142] Baker A. Pro-voice: how to keep listening when the world wants a fight. Oakland, CA: Berrett-Koehler Publishers, Inc, 2015.

[143] Laidman J. After decades of research, evaluating abortion’s effect still difficult. Toledo Blade, 22 1 2004, http://www.toledoblade.com/Politics/2004/01/22/After-decades-of-research-evaluating-abortion-s-effect-still-difficult.html

[144] Russo NF. Response from Div. 35 task force on reproductive issues. Monit Pyschology 2003; 34(4): 4.

[145] Major B Richards C Cooper ML et al Personal resilience, cognitive appraisals, and coping: an integrative model of adjustment to abortion. J Pers Soc Psychol 1998; 74(3): 735–752. 9523416 10.1037//0022-3514.74.3.735

[146] Coleman PK Boswell K Etzkorn K et al Women who suffered emotionally from abortion: a qualitative synthesis of their experiences. J Am Phys Surg 2017; 22(4): 113–118.

[147] Cohen BM. Embracing complexity in psychiatric diagnosis, treatment, and research. JAMA Psychiatry 2016; 511(7510): 421–427. 10.1001/jamapsychiatry.2016.2466 27829098

[148] Miller WB Pasta DJ Dean CL. Testing a model of the psychological consequences of abortion. In: Beckman LJ Harvey SM (eds) The new civil war: the psychology, culture, and politics of abortion. Washington, DC: American Psychological Association, 1998, pp. 235–267.

[149] Curley M. An explanatory model to guide assessment, risk and diagnosis of psychological distress after abortion. Open J Obstet Gynecol 2014; 4(15): 944–953.

[150] Hoekzema E Barba-Müller E Pozzobon C et al Pregnancy leads to long-lasting changes in human brain structure. Nat Neurosci 2016; 20: 287–296. 27991897 10.1038/nn.4458

[151] Cozzarelli C Sumer N Major B. Mental models of attachment and coping with abortion. J Pers Soc Psychol 1998; 74(2): 453–467. 9491587 10.1037//0022-3514.74.2.453

[152] Speckhard A Mufel N. Universal responses to abortion? Attachment, trauma, and grief responses in women following abortion. J Prenat Perinat Psychol 2003; 18: 3–37.

[153] Kaij L Malmquist A Nilsson Å. Psychiatric aspects of spontaneous abortion—II. The importance of bereavement, attachment and neurosis in early life. J Psychosom Res 1969; 13: 53–59. 5777000 10.1016/0022-3999(69)90019-1

[154] Allanson S Astbury J. Attachment style and broken attachments: violence, pregnancy, and abortion. Aust J Psychol 2001; 53: 146–151.

[155] Li J Laursen TM Precht DH et al Hospitalization for mental illness among parents after the death of a child. N Engl J Med 2005; 352(12): 1190–1196. 15788495 10.1056/NEJMoa033160

[156] Cacciatore J Defrain J Jones KLC When a baby dies: ambiguity and stillbirth. Marriage Fam Rev 2008; 44(4): 439–454.

[157] Keesee NJ Currier JM Neimeyer RA. Predictors of grief following the death of one’s child: the contribution of finding meaning. J Clin Psychol 2008; 64(10): 1145–1163. 18698614 10.1002/jclp.20502

[158] Rubin S Malkinson R. Parental response to child loss across the life cycle: clinical and research perspectives. In: Stroebe MS Hansson RO Stroebe W (eds) Handbook of bereavement research: consequences, coping, and care. Washington, DC: American Psychological Association, 2001, pp. 219–239.

[159] Kersting A Kroker K Steinhard J et al Complicated grief after traumatic loss: a 14-month follow up study. Eur Arch Psychiatry Clin Neurosci 2007; 257(8): 437–443. 17629729 10.1007/s00406-007-0743-1

[160] Speckhard AP Rue VP. Complicated mourning: dynamics of impacted post abortion grief. Pre-Peri-Natal Psychol J. 1993; 8(1): 5–32.

[161] Lang A Fleiszer A Duhamel F. Perinatal loss and parental grief: the challenge of ambiguity and disenfranchised grief. Omega 2011; 63(2): 183–196. 21842665 10.2190/OM.63.2.e

[162] Wijngaards-de Meij L Stroebe M Schut H et al Couples at risk following the death of their child: predictors of grief versus depression. J Consult Clin Psychol 2005; 73(4): 617–623. 16173849 10.1037/0022-006X.73.4.617

[163] Lundorff M Holmgren H Zachariae R et al Prevalence of prolonged grief disorder in adult bereavement: a systematic review and meta-analysis. J Affect Disord 2017; 212: 138–149. 28167398 10.1016/j.jad.2017.01.030

[164] Betz G Thorngren J. Ambiguous loss and the family grieving process. Fam J 2006; 14: 359–365.

[165] Rosenblatt P Burns L. Long-term effects of perinatal loss. J Fam Issues 1986; 7: 237–253.

[166] Kero A Lalos A. Ambivalence —a logical response to legal abortion: a prospective study among women and men. J Psychosom Obstet Gynaecol 2000; 21(2): 81–91. 10994180 10.3109/01674820009075613

[167] Bonanno GA Papa A Lalande K et al Grief processing and deliberate grief avoidance: a prospective comparison of bereaved spouses and parents in the United States and the People’s Republic of China. J Consult Clin Psychol 2005; 73(1): 86–98. 15709835 10.1037/0022-006X.73.1.86

[168] Wojnar DM Swanson KM Adolfsson A-S. Confronting the inevitable: a conceptual model of miscarriage for use in clinical Practice and research. Death Stud 2011; 35(6): 536–558. 24501829 10.1080/07481187.2010.536886

[169] Ney PG Fung T Wickett AR et al The effects of pregnancy loss on women’s health. Soc Sci Med 1994; 38(9): 1193–1200. 8016684 10.1016/0277-9536(94)90184-8

[170] Giannandrea SAM Cerulli C Anson E et al Increased risk for postpartum psychiatric disorders among women with past pregnancy loss. J Womens Health 2013; 22(9): 760–768. 10.1089/jwh.2012.4011 PMC3768229 24007380

[171] Speckhard AC Rue VM. Postabortion syndrome: an emerging public health concern. J Soc Issues 1992; 48(3): 95–119.

[172] Söderberg H Janzon L Sjöberg NO. Emotional distress following induced abortion: a study of its incidence and determinants among abortees in Malmö, Sweden. Eur J Obstet Gynecol Reprod Biol 1998; 79(2): 173–178. 9720837 10.1016/s0301-2115(98)00084-0

[173] Kero A Högberg U Lalos A. Wellbeing and mental growth-long-term effects of legal abortion. Soc Sci Med 2004; 58(12): 2559–2569. 15081205 10.1016/j.socscimed.2003.09.004

[174] Söderberg H Andersson C Janzon L et al Selection bias in a study on how women experienced induced abortion. Eur J Obstet Gynecol Reprod Biol 1998; 77(1): 67–70. 9550203 10.1016/s0301-2115(97)00223-6

[175] Poppema S Henderson M. Why I am an abortion doctor. New York: Prometheus Books, 1996, 266 pp.

[176] McCarthy C. Worst form of birth control hurts woman’s psyche. The Washington Post, 28 2 1971, p. B2.

[177] McCarthy C. The real anguish of abortions. The Washington Post, 5 2 1989, https://www.washingtonpost.com/archive/lifestyle/1989/02/05/the-real-anguish-of-abortions/b19f1b34-d561-415d-9974-1774c351cb5c/

[178] Jenkins N. Hardly any women regret having an abortion, a new study finds. Time, 14 7 2015, p. 10, http://time.com/3956781/women-abortion-regret-reproductive-health/ (accessed 28 December 2016).

[179] Rocca CH Kimport K Roberts SCM et al Decision rightness and emotional responses to abortion in the United States: a longitudinal study. PLoS ONE 2015; 10(7): e0128832. 26154386 10.1371/journal.pone.0128832 PMC4496083

[180] Dreaper J. Abortion “does not raise” mental health risk. BBC News, 2011, http://www.bbc.com/news/health-16094906 (accessed 28 December 2016).

[181] Reardon DC. Aborted women, silent no more. Chicago, IL: Loyola University Press, 1987, 373 pp.

[182] Dykes K Slade P Haywood A. Long term follow-up of emotional experiences after termination of pregnancy: women’s views at menopause. J Reprod Infant Psychol 2011; 29(1): 93–112.

[183] Reardon DC Ney PG. Abortion and subsequent substance abuse. Am J Drug Alcohol Abuse 2000; 26(1): 61–75. 10718164 10.1081/ada-100100591

[184] Reardon DC Cougle JR. Depression and unintended pregnancy in young women. BMJ 2002; 324(7345): 1097. PMC1123039 11991923

[185] Jones EF Forrest JD. Underreporting of abortion in surveys of U.S. women: 1976 to 1988. Demography 1992; 29(1): 113–126. 1547898

[186] Jones RK Kost K. Underreporting of induced and spontaneous abortion in the United States: an analysis of the 2002 National Survey of Family Growth. Stud Fam Plann 2007; 38(3): 187–197. 17933292 10.1111/j.1728-4465.2007.00130.x

[187] van Ditzhuijzen J ten Have M de Graaf R et al Psychiatric history of women who have had an abortion. J Psychiatr Res 2013; 47(11): 1737–1743. 23941742 10.1016/j.jpsychires.2013.07.024

[188] Biggs MA Upadhyay UD McCulloch CE et al Women’s mental health and well-being 5 years after receiving or being denied an abortion: a prospective, longitudinal cohort study. JAMA Psychiatry 2017; 74: 169–178. 27973641 10.1001/jamapsychiatry.2016.3478

[189] Ingraham C. 95 percent of women who’ve had an abortion say it was the right decision. The Washington Post, 14 7 2015, https://www.washingtonpost.com/news/wonk/wp/2015/07/14/95-percent-of-women-whove-had-an-abortion-say-it-was-the-right-decision/

[190] Adler NE. Sample attrition in studies of psychosocial sequelae of abortion: how great a problem? J Appl Soc Psychol 1976; 6(3): 240–259.

[191] Weisæth L. Importance of high response rates in traumatic stress research. Acta Psychiatr Scand 1989; 355: 131–137. 2624131

[192] Romans S. Asking the unanswerable: stymied again by the impossibility of sensible controls. Aust N Z J Psychiatry 2013; 47(9): 802–804. 23803898 10.1177/0004867413495319

[193] Fergusson DM Horwood LJ Boden JM. Abortion and mental health: a response to Romans and Steinberg. Aust N Z J Psychiatry 2013; 47(12): 1201–1203. 23928277 10.1177/0004867413500356

[194] Stotland NL Shrestha AD. More evidence that abortion is not associated with increased risk of mental illness. JAMA Psychiatry 2018; 75: 775–776. 29847615 10.1001/jamapsychiatry.2018.0838

[195] Karalis E Ulander V-M Tapper A-M et al Decreasing mortality during pregnancy and for a year after while mortality after termination of pregnancy remains high: a population-based register study of pregnancy-associated deaths in Finland 2001-2012. BJOG 2017; 124: 1115–1121. 28029218 10.1111/1471-0528.14484

[196] Gissler M Berg C Bouvier-Colle M-H et al Injury deaths, suicides and homicides associated with pregnancy, Finland 1987-2000. Eur J Public Health 2005; 15(5): 459–463. 16051655 10.1093/eurpub/cki042

[197] Shapiro GD Séguin JR Muckle G et al Previous pregnancy outcomes and subsequent pregnancy anxiety in a Quebec prospective cohort. J Psychosom Obstet Gynaecol 2017; 38(2): 121–132. 28079434 10.1080/0167482X.2016.1271979 PMC5383417

[198] Reardon DC. The embrace of the pro-abortion turnaway study. Wishful thinking? or willful deceptions? Linacre Q 2018; 85(3): 204–212. 30275603 10.1177/0024363918782156 PMC6161227

[199] Petersen R Moos M-K. Defining and measuring unintended pregnancy: issues and concerns. Womens Health Issues 1997; 7(4): 234–240. 9283277 10.1016/S1049-3867(97)00009-1

[200] Finer L Zolna M. Shifts in intended and unintended pregnancies in the United States, 2001–2008. Am J Public Health 2014; 104(Suppl. 1): S43–S48. 24354819 10.2105/AJPH.2013.301416 PMC4011100

[201] Russo N Zierk K. Abortion, childbearing, and women’s well-being. Prof Psychol Res 1992; 23: 269–280.

[202] Wells G Shea B O’Connell D et al The Newcastle-Ottawa Scale (NOS) for assessing the quality of nonrandomized studies in meta-analyses. Ottawa, ON, Canada: Department of Epidemiology and Community Medicine, University of Ottawa, http://www.ohri.ca/programs/clinical_epidemiology/oxford.htm

[203] Reardon DC Thorp JM. Pregnancy associated death in record linkage studies relative to delivery, termination of pregnancy, and natural losses: a systematic review with a narrative synthesis and meta-analysis. SAGE Open Med 2017; 5: 205031211774049. 10.1177/2050312117740490 PMC5692130 29163945

[204] Curley M Johnston C. The characteristics and severity of psychological distress after abortion among university students. J Behav Heal Serv Res 2013; 40(3): 279–293. 10.1007/s11414-013-9328-0 23576135

[205] Galbally M Lewis AJ Ijzendoorn Mv et al The role of oxytocin in mother-infant relations: a systematic review of human studies. Harv Rev Psychiatry 2011; 19(1): 1–14. 21250892 10.3109/10673229.2011.549771

[206] Kleinman JC Kovar MG Feldman JJ et al A comparison of 1960 and 1973-1974 early neonatal mortality in selected states. Am J Epidemiol 1978; 108(6): 454–469. 736025 10.1093/oxfordjournals.aje.a112644

[207] Grace JT. Development of maternal-fetal attachment during pregnancy. Nurs Res 2017; 38(4): 228–232. 2748357

[208] Munk-Olsen T Gasse C Laursen TM. Prevalence of antidepressant use and contacts with psychiatrists and psychologists in pregnant and postpartum women. Acta Psychiatr Scand 2012; 125(4): 318–324. 22118213 10.1111/j.1600-0447.2011.01784.x

[209] Munk-Olsen T Maegbaek ML Johannsen BM et al Perinatal psychiatric episodes: a population-based study on treatment incidence and prevalence. Transl Psychiatry 2016; 6(10): e919. 27754485 10.1038/tp.2016.190 PMC5315550

[210] Munk-Olsen T Pedersen HS Laursen TM et al Use of primary health care prior to a postpartum psychiatric episode. Scand J Prim Health Care 2015; 33(2): 127–133. 26174691 10.3109/02813432.2015.1041832 PMC4834500

[211] Kumar R Robson K. Previous induced abortion and ante-natal depression in primiparae: preliminary report of a survey of mental health in pregnancy. Psychol Med 1978; 8(4): 711–715. 724880 10.1017/s0033291700018912

[212] McCarthy F Moss-Morris R Khashan A et al Previous pregnancy loss has an adverse impact on distress and behaviour in subsequent pregnancy. BJOG 2015; 122(13): 1757–1764. 25565431 10.1111/1471-0528.13233

[213] Bradley CF. Abortion and subsequent pregnancy. Can J Psychiatry 1984; 29(6): 494–498. 6488130 10.1177/070674378402900608

[214] Kitamura T Shima S Sugawara M et al Psychological and social correlates of the onset of affective disorders among pregnant women. Psychol Med 1993; 23(4): 967–975. 8134520 10.1017/s003329170002643x

[215] Devore NE. The relationship between previous elective abortions and postpartum depressive reactions. JOGN Nurs 1979; 8(4): 237–240. 258266 10.1111/j.1552-6909.1979.tb00834.x

[216] Huang Z Hao J Su P et al The impact of prior abortion on anxiety and depression symptoms during a subsequent pregnancy: data from a population-based cohort study in China. Bull Clin Psychopharmacol 2012; 22(11): 51–58.

[217] Munk-Olsen T Bech BH Vestergaard M et al Psychiatric disorders following fetal death: a population-based cohort study. BMJ Open 2014; 4: e005187. 10.1136/bmjopen-2014-005187 PMC4054628 24907247

[218] Waqas A Raza N Lodhi HW et al Psychosocial factors of antenatal anxiety and depression in Pakistan: is social support a mediator? PLoS ONE 2015; 10(1): e0116510. 25629925 10.1371/journal.pone.0116510 PMC4309576

[219] Ali NS Azam IS Ali BS et al Frequency and associated factors for anxiety and depression in pregnant women: a hospital-based cross-sectional study. Sci World J 2012; 2012: 653098. 10.1100/2012/653098 PMC3354685 22629180

[220] Ajinkya S Jadhav PR Srivastava NN. Depression during pregnancy: prevalence and obstetric risk factors among pregnant women attending a tertiary care hospital in Navi Mumbai. Ind Psychiatry J 2013; 22(1): 37–40. 24459372 10.4103/0972-6748.123615 PMC3895310

[221] Faisal-Cury A Rossi Menezes P. Prevalence of anxiety and depression during pregnancy in a private setting sample. Arch Womens Ment Health 2007; 10(1): 25–32. 17187166 10.1007/s00737-006-0164-6

[222] Chojenta C Harris S Reilly N et al History of pregnancy loss increases the risk of mental health problems in subsequent pregnancies but not in the postpartum. PLoS ONE 2014; 9(4): e95038. 24733508 10.1371/journal.pone.0095038 PMC3986356

[223] Al-Shami N Moawed SEA Identification of factors associated with postpartum depression among Saudi females in Riyadh City, 2010, http://repository.ksu.edu.sa/jspui/handle/123456789/19373 (accessed 3 January 2017).

[224] Räisänen S Lehto SM Nielsen HS et al Fear of childbirth predicts postpartum depression: a population-based analysis of 511 422 singleton births in Finland. BMJ Open 2013; 3(11): e004047. 10.1136/bmjopen-2013-004047 PMC3845069 24293208

[225] Montmasson H Bertrand P Perrotin F et al Facteurs prédictifs de l’état de stress post-traumatique du postpartum chez la primipare. J Gynécologie Obs Biol la Reprod 2012; 41(6): 553–560. 10.1016/j.jgyn.2012.04.010 22622194

[226] Kumar R Robson KM. A prospective study of emotional disorders in childbearing women. Br J Psychiatry 1984; 144: 35–47. 6692075 10.1192/bjp.144.1.35

[227] Reardon DC. Lack of pregnancy loss history mars depression study. Acta Psychiatr Scand 2012; 126(2): 155. 22616564 10.1111/j.1600-0447.2012.01880.x

[228] Afable-Munsuz A Speizer I Magnus JH et al A positive orientation toward early motherhood is associated with unintended pregnancy among New Orleans youth. Matern Child Health J 2006; 10(3): 265–276. 16382331 10.1007/s10995-005-0049-8

[229] Ney PG. Some real issues surrounding abortion, or, the current practice of abortion is unscientific. J Clin Ethics 1993; 4(2): 179–180. 8334286

[230] Ulene V. Life with Lasik: a closer look. Los Angeles Times, 16 6 2008, http://articles.latimes.com/2008/jun/16/health/he-themd16

[231] Murphy JF O’Driscoll K. Therapeutic abortion: the medical argument. Ir Med J 1982; 75(8): 304–306. 7129852

[232] Reardon DC Strahan TW Thorp JM et al Deaths associated with abortion compared to childbirth —a review of new and old data and the medical and legal implications. J Contemp Health Law Policy 2004; 20(2): 279–327. 15239361

[233] Calhoun BC. Induced abortion and risk of later premature births. J Am Phys Surg 2003; 8(2): 46–49.

[234] 8th Cir. (en banc). Planned Parenthood Minn., N.D., S.D. v. Rounds, 2012.

[235] United Nations. Beijing declaration and platform for action. In: Adopted at the fourth world conference on women, Beijing, China, 4–15 September 1995.

[236] Bradford-Hill A. The environment and disease: association or causation? Proc R Soc Med 1965; 58: 295–300. 14283879 10.1177/003591576505800503 PMC1898525

[237] Biggs MA Neuhaus JM Foster DG. Mental health diagnoses 3 years after receiving or being denied an abortion in the United States. Am J Public Health 2015; 105(12): 2557–2563. 26469674 10.2105/AJPH.2015.302803 PMC4638270

[238] Sriarporn P Turale S Lordee N et al Support program for women suffering grief after termination of pregnancy: a pilot study. Nurs Heal Sci 2017; 19(1): 75–80. 10.1111/nhs.12307 27620532

[239] Ney PG Ball K Sheils C. Results of group psychotherapy for abuse, neglect and pregnancy loss. Curr Womens Health Rev 2010; 6(4): 332–340.

[240] Jaramillo S. Mending broken lives: post-abortion healing. Virginia Beach, VA: Regent University, 2017.

[241] Artist hanged herself after aborting her twins. The Telegraph, 22 2 2008, http://www.telegraph.co.uk/news/uknews/1579455/Artist-hanged-herself-after-aborting-her-twins.html (accessed 31 March 2016).

[242] Hogan MC Foreman KJ Naghavi M et al Maternal mortality for 181 countries, 1980-2008: a systematic analysis of progress towards Millennium Development Goal 5. Lancet 2010; 375(9726): 1609–1623. 20382417 10.1016/S0140-6736(10)60518-1

[243] Herman JL. Trauma and recovery: the aftermath of violence —from domestic abuse to political terror. New York: Basic Books, 1992.

[244] Duhaime L. Thin skull rule definition. Duhaime’s Law Dictionary, 2016, http://www.duhaime.org/LegalDictionary/T/ThinSkullRule.aspx

[245] Needle RB Walker LE. Abortion counseling: a clinician’s guide to psychology, legislation, politics, and competency. New York, NY: Springer Publishing Company, 2007.

[246] Gissler M Hemminki E. Pregnancy-related violent deaths. Scand J Public Health 1999; 27: 54–55. 10847672

[247] Taft AJ Watson LF Lee C. Violence against young Australian women and association with reproductive events: a cross-sectional analysis of a national population sample. Aust N Z J Public Health 2004; 28(4): 324–329. 15704695 10.1111/j.1467-842x.2004.tb00438.x

[248] Russo NF. Abortion, unwanted childbearing, and mental health. Salud Ment 2014; 37(4): 283–291.

[249] Shuping M. Risk factors. In: MacNair RM (ed.) Peace psychology perspectives on abortion. Kansas City, MO: Feminism & Nonviolence Studies Association, 2016, pp. 94–114.

[250] Reardon D Makimma J Sobie A (eds). Victims and victors: speaking out about their pregnancies, abortions, and children resulting from sexual assault. Springfield, IL: Acorn Books, 2000, 192 pp.

[251] Robinson GE Stotland NL Russo NF et al Is there an “abortion trauma syndrome”? Critiquing the evidence. Harv Rev Psychiatry 2009; 17(4): 268–290. 19637075 10.1080/10673220903149119

[252] Stotland NL. The myth of the abortion trauma syndrome. JAMA J Am Med Assoc 1992; 268(15): 2078–2079. 1404747

[253] Sentilhes L Maillard F Brun S et al Risk factors for chronic post-traumatic stress disorder development one year after vaginal delivery: a prospective, observational study. Sci Rep 2017; 7(1): 8724. 28821837 10.1038/s41598-017-09314-x PMC5562814

[254] Mahmoodi Z Dolatian M Shaban Z et al Correlation between kind of delivery and posttraumatic stress disorder. Ann Med Health Sci Res 2016; 6(6): 356–361. 28540103 10.4103/amhsr.amhsr_397_15 PMC5423335

[255] Dikmen-Yildiz P Ayers S Phillips L. Longitudinal trajectories of post-traumatic stress disorder (PTSD) after birth and associated risk factors. J Affect Disord 2018; 229: 377–385. 29331697 10.1016/j.jad.2017.12.074

[256] Lee C Slade P. Miscarriage as a traumatic event: a review of the literature and new implications for intervention. J Psychosom Res 1996; 40(3): 235–244. 8861119 10.1016/0022-3999(95)00579-x

[257] Farren J Jalmbrant M Ameye L et al Post-traumatic stress, anxiety and depression following miscarriage or ectopic pregnancy: a prospective cohort study. BMJ Open 2016; 6(11): e011864. 10.1136/bmjopen-2016-011864 PMC5129128 27807081

[258] Krosch DJ Shakespeare-Finch J. Grief, traumatic stress, and posttraumatic growth in women who have experienced pregnancy loss. Psychol Trauma 2017; 9(4): 425–433. 27607765 10.1037/tra0000183

[259] Coleman PK Coyle CT Rue VM. Late-term elective abortion and susceptibility to posttraumatic stress symptoms. J Pregnancy 2010; 2010: 130519. 21490737 10.1155/2010/130519 PMC3066627

[260] Hamama L Rauch SA Sperlich M et al Previous experience of spontaneous or elective abortion and risk for posttraumatic stress and depression during subsequent pregnancy. Depress Anxiety 2010; 27(8): 699–707. 20577979 10.1002/da.20714 PMC2939862

[261] Kersting A Kroker K Steinhard J et al Psychological impact on women after second and third trimester termination of pregnancy due to fetal anomalies versus women after preterm birth-a 14-month follow up study. Arch Womens Ment Health 2009; 12(4): 193–201. 19266250 10.1007/s00737-009-0063-8

[262] Kelly T Suddes J Howel D et al Comparing medical versus surgical termination of pregnancy at 13-20 weeks of gestation: a randomised controlled trial. BJOG 2010; 117(12): 1512–1520. 20860598 10.1111/j.1471-0528.2010.02712.x

[263] Borins EFM Forsythe PJ Past trauma and present functioning of patients attending a women’s psychiatric clinic. Am J Psychiatry 1985; 142(4): 460–463. 3976919 10.1176/ajp.142.4.460

[264] Fisch RZ Tadmor O. Iatrogenic post-traumatic stress disorder. Lancet 1989; 2(8676): 1397. 10.1016/s0140-6736(89)92007-2 2574339

[265] Vukelić J Kapamadzija A Kondić B. Investigation of risk factors for acute stress reaction following induced abortion. Med Pregl 2018; 63(5–6): 399–403. 10.2298/mpns1006399v 21186554

[266] Wallin Lundell I Sundström Poromaa I Ekselius L et al Neuroticism-related personality traits are associated with posttraumatic stress after abortion: findings from a Swedish multi-center cohort study. BMC Womens Health 2017; 17(1): 96. 28969621 10.1186/s12905-017-0417-8 PMC5625823

[267] Zulčić-Nakić V Pajević I Hasanović M et al Psychological problems sequalae in adolescents after artificial abortion. J Pediatr Adolesc Gynecol 2012; 25(4): 241–247. 22840934 10.1016/j.jpag.2011.12.072

[268] Suliman S Ericksen T Labuschgne P et al Comparison of pain, cortisol levels, and psychological distress in women undergoing surgical termination of pregnancy under local anaesthesia versus intravenous sedation. BMC Psychiatry 2007; 7: 24. 17565666 10.1186/1471-244X-7-24 PMC1899490

[269] Rousset C Brulfert C Séjourné N et al Posttraumatic stress disorder and psychological distress following medical and surgical abortion. J Reprod Infant Psychol 2011; 29(5): 506–517.

[270] Gravensteen IK Helgadóttir LB Jacobsen E-M et al Women’s experiences in relation to stillbirth and risk factors for long-term post-traumatic stress symptoms: a retrospective study. BMJ Open 2013; 3(10): e003323. 10.1136/bmjopen-2013-003323 PMC3808779 24154514

[271] Ryan GL Mengeling MA Booth BM et al Voluntary and involuntary childlessness in female veterans: associations with sexual assault. Fertil Steril 2014; 102(2): 539–547. 24875400 10.1016/j.fertnstert.2014.04.042

[272] Fergusson DM. Abortion and mental health. Psychiatr Bull 2008; 32(9): 321–324.

[273] Great Britain Commission of Inquiry into the Operation and Consequences of The Abortion Act. The physical and psycho-social effects of abortion on women. London: Great Britain, Parliament, House of Lords, Commission of Inquiry into the Operation and Consequences of The Abortion Act, 1994.

[274] National Collaborating Centre for Mental Health. Induced abortion and mental health systematic review consultation—6 April to 29 June 2011 comments and responses. London: Academy of Medical Royal Colleges, 2011, https://www.scribd.com/document/327608591/Induced-Abortion-and-Mental-Health-Systematic-Review-Consultation-6-April-to-29-June-2011-Comments-and-Responses

[275] Coleman P Speckhard A Ney P et al NCCMH review. AbortionRisks.org, 2017, http://abortionrisks.org/index.php?title=NCCMH_Review (accessed 8 February2017).

[276] Morey RD Chambers CD Etchells PJ et al The Peer Reviewers’ Openness Initiative: incentivizing open research practices through peer review. R Soc Open Sci 2016; 3(1): 150547. 26909182 10.1098/rsos.150547 PMC4736937

[277] Schofield PN Bubela T Weaver T et al Post-publication sharing of data and tools. Nature 2009; 461(7261): 171–173. 19741686 10.1038/461171a PMC6711854

[278] American Psychological Association. Ethical principles of psychologists and code of conduct, 2010, http://www.apa.org/ethics/code/index.aspx#8_14 (accessed 2 February 2017).

[279] Wicherts JM Borsboom D Kats J et al The poor availability of psychological research data for reanalysis. Am Psychol 2006; 61(7): 726–728. 17032082 10.1037/0003-066X.61.7.726

[280] Vanpaemel W Vermorgen M Deriemaecker L et al Are we wasting a good crisis? The availability of psychological research data after the storm. Collabra 2015; 1(1): 3.

[281] Naik G. Peer-review activists push psychology journals towards open data. Nature 2017; 543: 161. 28277531 10.1038/nature.2017.21549

[282] Reardon DC Coleman PK Munk-Olsen et al. AbortionRisks.org, 2017, http://abortionrisks.org/index.php?title=Munk-Olsen_et_al (accessed 2 February 2017).

[283] Munk-Olsen T Laursen TM Meltzer-Brody S et al Psychiatric disorders with postpartum onset: possible early manifestations of bipolar affective disorders. Arch Gen Psychiatry 2012; 69: 428–434. 22147807 10.1001/archgenpsychiatry.2011.157

[284] Munk-Olsen T Agerbo E. Does childbirth cause psychiatric disorders? A population-based study paralleling a natural experiment. Epidemiology 2015; 26(1): 79–84. 25322321 10.1097/EDE.0000000000000193 PMC4250401

[285] Blackmore ER Côté-Arsenault D Tang W et al Previous prenatal loss as a predictor of perinatal depression and anxiety. Br J Psychiatry 2011; 198(5): 373–378. 21372060 10.1192/bjp.bp.110.083105 PMC3084335

[286] Räisänen S Lehto SM Nielsen HS et al Risk factors for and perinatal outcomes of major depression during pregnancy: a population-based analysis during 2002-2010 in Finland. BMJ Open 2014; 4(11): e004883. 10.1136/bmjopen-2014-004883 PMC4244456 25398675

[287] Marengo E Martino DJ Igoa A et al Unplanned pregnancies and reproductive health among women with bipolar disorder. J Affect Disord 2015; 178: 201–205. 25827504 10.1016/j.jad.2015.02.033

